# Contribution of bacterial pathogens to evoking serological disease markers and aggravating disease activity in rheumatoid arthritis

**DOI:** 10.1371/journal.pone.0190588

**Published:** 2018-02-06

**Authors:** Kuniaki Terato, Takaki Waritani, Richio Fukai, Hiroshi Shionoya, Hiroshi Itoh, Kou Katayama

**Affiliations:** 1 Department of Research and Development, Chondrex Inc. Redmond, WA, United States of America; 2 Fukai Pharmacy, Asahikawa, Hokkaido, Japan; 3 Research Lab Section 5, Asama Chemicals Inc. Chiyoda, Tokyo, Japan; 4 Department of Orthopedic Surgery, Asahikawa Medical University, Asahikawa, Japan; 5 Katayama Orthopedic Rheumatology Clinic, Asahikawa, Hokkaido, Japan; Universite Paris-Sud, FRANCE

## Abstract

Commensal bacteria and their pathogenic components in the gastrointestinal tract and oral cavity may play pathological roles in autoimmune diseases. To study the possible involvement of bacterial pathogens in autoimmune diseases, IgG and IgA antibodies against pathogenic components produced by three strains of commensal bacteria, *Escherichia coli*-lipopolysaccharide (*E*. *coli*-LPS), *Porphyromonas gingivalis*-LPS (Pg-LPS) and peptidoglycan polysaccharide (PG-PS) from *Streptococcus pyogenes*, were determined by an improved ELISA system for sera from two groups of patients with rheumatoid arthritis (RA), who met rapid radiographic progression (RRP) criteria and non-RRP, and compared to normal (NL) controls. Antibody responses to these bacterial pathogens are unique and consistent in individuals, and no fundamental difference was observed between RA and NL controls. Despite the similar antibody responses to pathogens, lower IgG or higher IgA and consequent higher IgA/IgG antibody ratio among the patients with RA related to disease marker levels and disease activity. Peculiarly, the IgA/IgG anti-Pg-LPS antibody ratio resulted from lower IgG and higher IgA antibody responses to Pg-LPS strongly correlated not only with rheumatoid factor (RF), but also correlated with erythrocyte sedimentation rate (ESR), C-reactive protein (CRP) and disease activity score of 28 joints with ESR (DAS28-ESR) in the RRP group. In contrast, the IgA/IgG anti-*E*. *coli*-LPS and anti-PG-PS antibody ratio correlated or tended to correlate with RF, ESR, CRP, and DAS28-ESR in the non-RRP group, whereas either the IgG or IgA anti-Pg-LPS antibody levels and consequent IgA/IgG anti-Pg-LPS antibody ratio did not correlate with any clinical marker levels in this group. Notably, anti-circular-citrullinated peptide (CCP) antibody levels, which did not correlate with either IgG or IgA antibody levels to any pathogens, did not correlate with severity of arthritis in both RRP and non-RRP. Taken together, we propose that multiple environmental pathogens, which overwhelm the host antibody defense function, contribute independently or concomitantly to evoking disease makers and aggravating disease activity, and affect disease outcomes.

**Trial registration**: UMIN CTR UMIN000012200

## Introduction

A variety of chronic and progressive inflammatory diseases are classified as autoimmune diseases. To search for disease-causative pathogens, numerous studies on antibody responses to putative pathogens were conducted, and commonly concluded that antibodies against putative pathogens are higher, or cross-react with major histocompatibility complex (MHC) molecules or other autologous components such as collagen in rheumatoid arthritis (RA) [1], and cardiac myosin in rheumatic heart disease [2]. Thus, autoimmunity in rheumatic diseases is thought to be induced by microbial infections via cross-reactivity or molecular mimicry [3].

Based on these concepts, antibody responses to variety of potential pathogenic environmental agents have been studied: for example, antibody responses to *Escherichia coli (E*. *coli)*, *Klebsiella pneumonia*, *Proteus mirabilis*, *Serratia marcescebs* [3–8], *Porphyromonas gingivalis* (*P*. *gingivalis*) [6, 9], and lipopolysaccharides (LPS) produced by commensal bacteria [10]. Unfortunately, these studies were conducted using immunoassay systems such as radioimmunoassay (RIA) and enzyme-linked immunosorbent assay (ELISA) without considering the intense background (BG) noise reaction caused by hydrophobic binding of immunoglobulins and immune-complexes in sample specimens to plastic surfaces. Consequently, misinterpretation of serological antibody assay data, which are largely influenced by the strong BG noise reaction and other false positive reactions, has led to uncertain conclusions and misunderstandings as discussed in detail [11–14].

Meanwhile, a growing body of research has indicated a potential association between intestinal bacteria and autoimmune diseases [15–22]. More specifically, dysbiosis, or imbalance of intestinal bacterial flora in RA was suggested by many studies on possible composition changes in intestinal microbes [23–28]. Especially, Scher [29] clearly showed that an increase of *Prevotella copri* (*P*. *copri*) and a decrease of *Bacteroides* populations in stool were associated with disease in new-onset, untreated patients with RA. These observations and hypotheses were supported by a variety of studies on potential disease-causative pathogens in animal models. For example, possible involvement of bacterial cell wall components in the pathogenesis of RA was suggested by studies on the arthropathic properties of bacterial cell wall polymers such as peptidoglycan-polysaccharide (PG-PS) from *Streptococcus pyrogens* (*S*. *pyrogen*) [30–32], *S*. *faecium* and other normal flora [33]. Furthermore, striking effects of a variety of bacterial toxins, such as *Mycoplasma arthritidis* mitogen (MAM) [34, 35], *E*. *coli*-LPS [35, 36], and *staphylococcus* super-antigen B (SEB) [37], in triggering and exacerbating arthritis were shown in mouse collagen-induced arthritis (CIA) and collagen antibody-induced arthritis (CAIA) models.

Likewise, increasing evidence suggests a possible link between RA and periodontal infectious diseases caused by *P*. *gingivalis* [38–40] and *Aggregatibacter actinomycetemcomitans* (A. *actinomycetemcomitans*) [41]. This prediction is supported by studies in a mouse CIA model. Indeed, *P*. *gingivalis* infection significantly facilitates the development and progression of arthritis, including bone absorption and cartilage destruction [42–45]. Based on these observations, it is assumed that some of these pathogens are implicated in enhancing and perpetuating inflammatory arthritis, leading to severe joint damage, such as rapid radiographic progression (RRP) observed in a subset of RA patients [46–49].

In addition to potential pathogenic agents, it is also important to consider possible disorders in the host that increase susceptibility to disease-causative pathogens. For example, if the immunological and mechanical barriers of the gastrointestinal tract are disturbed by intestinal disease, the aging process, or other factors, it is highly likely that abnormal amounts of mimic antigens and bacteria-derived pathogens will be absorbed from the gastrointestinal tract. In fact, it was clearly demonstrated that bacterial toxins absorbed from the intestine are directly implicated in the development of arthritis in animal models. For example, oral administration of *E*. *coli*-LPS relapses arthritis at a late stage of CIA [36]. Similarly, a single oral administration of *E*. *coli*-LPS increases serum IL-6 levels within 2 hours, and triggers CAI within 48 hours in mice older than 8 months, but not in young mice [50]. Furthermore, long term oral administration of heterologous type II collagen (CII), *E*. *coli-*LPS, or a combination of these induces mild, but chronic arthritis in DBA/1 mice [51]. More importantly, *E*. *coli*-LPS alone induces severe destructive arthritis in mice whose immune system was disturbed by a long term oral administration of a combination of indomethacin and ovoinhibitor [51].

From this standpoint, we hypothesized that lowered immune defense function or dysbiosis in the intestinal and oral environments may be the fundamental disorder in autoimmune disease, and consequently, patients are consistently exposed to excessive amounts of a variety of potential disease-causative bacterial pathogens [50, 52]. To test this hypothesis, IgG and IgA antibody responses against pathogenic components of three strains of bacteria were determined for sera from two groups of RA patients, who met RRP criteria and non-RRP, and normal (NL) controls by ELISA using the ChonBlock^TM^ buffer system, which prevents virtually all types of non-specific reactions involved the indirect ELISA as reported [13]. Antibody responses against individual pathogens were analyzed for potential correlation with serological disease maker levels and disease activity. In this study, we found that various types of bacterial pathogens, which overwhelm IgG and IgA antibody responses, may play critical pathological roles independently or concomitantly. Importantly, disease outcomes differ noticeably depending on the types of pathogens dominantly involved.

## Patients and methods

### RA patients and clinical assessment

Two hundred sixty-four patients with RA were enrolled in a clinical study to examine the effects of non-biological disease-modifying anti-rheumatic drugs (DMARDs) and biological DMARDs (biologics) on intestinal immunity in patients with RA at Katayama Orthopedic Rheumatology Clinic (Trial Registration Number: UMIN000012200 approved by Asahikawa Medical University Ethics Committee). The study purposes and procedures were provided in written form, and informed consent was obtained from all patients and normal subjects before performing any study procedure according the Declaration of Helsinki. Patients with RA were diagnosed based on the American College of Rheumatology (ACR) 1987 revised criteria [53]. Clinical disease activity was assessed by measuring tender 28 joint count (TJC28), swollen 28 joint count (SJC28), disease activity score of 28 joints with erythrocyte sedimentation rate (DAS28-ESR), and visual analogue scale of patients’ global estimate for RA (pVAS). In addition, radiographic films of patients’ hands and feet were assessed by modified total sharp score (mTSS) [54] at first serum collection, and compared with those obtained 1 year before, to determine the annual change of mTSS (∆mTSS/y). The patients whose mTSS value increased ≥5 units over a 1 year period were classified as RRP, since the complete destruction of one joint during a 1 year period corresponds to an increase of 5 units [49, 54]. Furthermore, the presence of osteitis and precise osteitis area were determined by magnetic resonance imaging (MRI) (HITACHI 0.3T Open MRI system AIRIS Elite) using Short T1 Inversion Recovery (STIR) method as reported [55]. In this test, we confirmed that moderate to severe osteitis was present in small joints (finger, wrist, foot) in these patients as described [56, 57]. In addition, in the patients with rapid progression of middle to large joints, regardless of the ∆mTSS/y of small joints, severe osteitis was observed at all small, middle and large joints. Therefore, these patients were included in the RRP group, because we considered that rapid progression of middle to large joints is one of the features of this disease phenotype, and should be distinguished from other RA phenotypes. Accordingly, it was confirmed that no patients in the non-RRP group have either apparent osteitis or destructive progression at middle to large joints.

### Blood and serum samples

Blood and serum samples were collected from all patients under treatment with current drugs before switching to other therapeutics to obtain the baseline data; ESR, C-reactive protein (CRP), matrix metalloproteinase-3 (MMP3), IgM-RF (RF), lymphocytes, white blood cells (WBC), red blood cells (RBC) and hemoglobin (Hb). One aliquot of serum sample for each patient was kept at -20°C, and assayed for serum tumor necrosis factor-α (TNF-α), interleukin 6 (IL-6), and antibodies against bacterial pathogens and circular-citrullinated peptide (CCP) For the purposes of this study, the baseline data from the selected patients, who were not accompanied by severe comorbidity and currently not treated with biological therapeutics, were collected for analysis in this study. Consequently, complete baseline data were available for 54 patients with RRP and 101 patients with non-RRP as shown in Table 1. Sera from 38 healthy NL controls (Age: 42±14) were used as a reference.

**Table 1:** Baseline demographics of RA patients in RRP and Non-RRP groups.

		RRP (n = 54)	Non-RRP (n = 101)	Normal (n = 38)
Age		62±14	66±11	42±14*
Sex	Female/Male	50/4	86/15	16/22
Duration (Month)		48 (23–151)	116 (52–174)	/
Therapeutics	AU	0	2 (2.0%)	/
	BUC	5 (9.3%)	11 (10.9%)	/
	Celecoxib	0	1 (1.0%)	/
	LEF	0	3 (3.0%)	/
	Minocyclin	0	1 (1.0%)	/
	Mizoribine	1 (1.9%)	0	/
	MTX	38 (70.4%)	65 (64.4%)	
	SASP	3 (5.6%)	16 (15.8%)	/
	TAC	7 (13.0%)	2 (2.0%)	/
Disease Activity Score	SJC28	5 (3–7.5)	5 (3–7)	/
	TJC28	2 (1–7)	5 (2–8)	/
	DAS28-ESR	4.4 ± 1.4	4.7 ± 1.2	/
	pVAS	48 (26–66)	40 (30–65)	/
Serological Disease Markers	CRP (mg/dl)	0.7 (0.1–1.9)	0.5 (0.1–1.9)	/
	ESR (mm/hour)	22.5 (12.8–44)	27 (12–46)	/
	IgM-RF (IU/ml)	84 (24.1–219)	51 (11.5–167)	/
	Anti-CCP Ab (units/ml)	3.7 (0.8–8.7)	1.1 (0.2–20)	0.08 (0.047–0.099)
Inflammatory Markers	TNF-α (pg/ml)	1.4 (0.9–2.2)	1.3 (1.1–1.9)	/
	IL-6 (pg/ml)	2.5 (1.0–8.2)	6.1(2.3–16.5)	/
	MMP3 (ng/ml)	128 (74.6–240	120 (64.4–217)	/
Hematological Markers	RBC (10^6^/μl)	415±32.6	417±48	/
	WBC (count/μl)	7211±2687	6478±1757	/
	Lymphocytes (count/μl)	1539 ± 481	1442 ± 476	/
	Hb (g/dl)	12.3 ± 1.4	12.5 ± 1.4	/
Radiographic	mTSS	37 (14–68)	23 (12–76)	/
Assessment	ΔmTSS/year	5.8 (2.2–11)**	0.0 (0–0.8)	/

*: P<0.05,

**: P< 0.0001 by Wilcoxon signed-rank test,

AU: Auranofin, BUC: Bucillamine, LEF: Leflunomide, MTX: Methotrexate, SASP: Salzosulfapyridine, TAC: Tacrolimus, RRP: Rapid radiographic progression, mTSS: modified total sharp scoring, ∆mTSS/y: annual change of mTSS during a 1 year. Clinical diagnostic data, which were distributed non-symmetrically, are shown as median (interquartile range), and others are shown as mean ± SD.

### Antibody assay

IgG and IgA antibody responses to three bacterial pathogens, LPS from *E*. *coli* O-111B4 (*E*. *coli*-LPS) (ultra-pure *E*. *coli*-LPS, List Biological Laboratories, Campbell, CA), LPS from *P. gingivalis* (Pg-LPS) (InvivoGen, San Diego, CA), PG-PS from *S*. *pyogenes* (Lee Laboratories, Grayson, GA), and CCP synthesized at Biosynthesis (Lewisville, TX) were assayed by ELISA using the ChonBlock^TM^ buffer system as described in detail [13, 58]. A detailed ELISA protocol is available in the attached supplemental information (S1 Appendix). Briefly, human sera from both patients with RA and NL controls were diluted with ChonBlock^TM^ blocking/sample dilution buffer as follows: for IgG and IgA antibodies against *E*. *coli*-LPS: 1/1,000 & 1/1,000 (for both IgG and IgA), Pg-LPS: 1/10,000 & 1/500, PG-PS: 1/20,000 & 1/10,000, and IgG antibodies against CCP: 1/500. Antibody levels were determined by comparing to standards prepared from normal sera, and expressed as x10^3^ units /ml. The highest dose of standard was adjusted to give an OD at 450nm of 2.8±0.1, and defined as 32 units/ml.

To determine the comprehensive immune function of individual patients, IgG and IgA antibody titers against individual pathogens were standardized by dividing with a mean antibody value of NL controls, and defined as IgG and IgA Antibody Index, respectively. The sum of index values calculated based on antibody levels against “*E*. *coli*-LPS and Pg-LPS “was defined as Index 1, “*E*. *coli*-LPS plus PG-PS” as Index 2, “*E*. *coli*-LPS plus Pg-LPS and PG-PS” as Index 3.

### Cytokine assay

Serum levels of TNF-α and IL-6 were assayed by high sensitivity human ELISA kits (Quantikine HS ELISA, R&D Systems, MIN, USA), and shown as pg/ml.

### Statistical analysis

Serum IgG and IgA antibody levels against all pathogens tested indicated non-normal distributions among NL, RRP and non-RRP groups. Accordingly, the statistical relationships between antibody levels, serological marker levels and disease activity were analyzed by Spearman’s non-parametric rank correlation analysis, and expressed as Spearman’s rank correlation coefficient “ρ” (JMP10 SAS Institute Inc., Cary, NC, USA). Similarly, the relationship between IgG and IgA antibody responses to individual pathogens was also determined by Spearman’s non-parametric rank correlation analysis. Data were shown as nominal p-values without adjustment for multiple testing. To demonstrate the relationship between two variables in log-log scale scattered graphs, 0 value was converted to 0.01 for practical convenience.

## Results

### 1. Characterization of IgG and IgA antibody responses to bacterial pathogens in NL, RRP, and non-RRP groups

Clinical demographics of 54 RRP and 101 non-RRP patients were similar, and 38 (70.4%) patients with RRP and 65 (60.4%) patients with non-RRP were treated with methotrexate (MTX) (Table 1).

**Fig 1:**
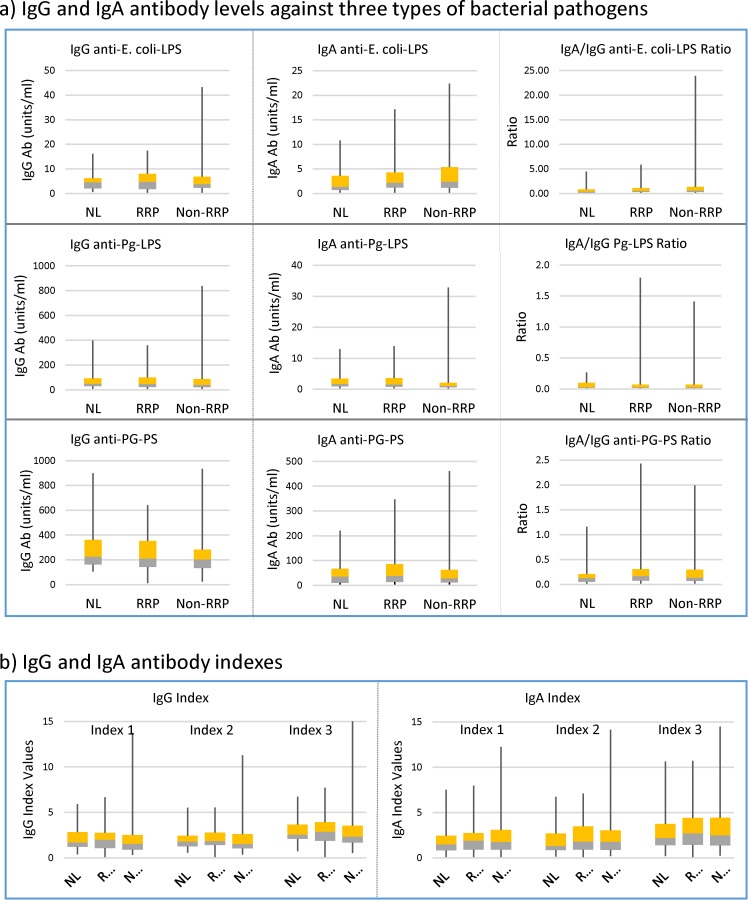
Comparison of antibody responses to potential pathogenic environmental agents between RA and NL controls. IgG and IgA antibody levels against *E*. *coli*-LPS, Pg-LPS and PG-PS were determined in sera from 38 NL controls, 54 patients with RRP and 101 patients with non-RRP (a). IgG and IgA antibody levels of individual patients were divided by the average values of NL controls, and shown as IgG and IgA index values (b). Data are shown as median and interquartile range (IQR). *E*. *coli*: *Escherichia coli*, Pg: *Porphyromonas gingivalis*, LPS: lipopolysaccharide, PG-PS: peptidoglycan polysaccharide from *Streptococcus pyogenes*, RRP: rapid radiographic progression, NOTE: Index 1: sum of anti-*E*. *coli*-LPS + anti-Pg-LPS, Index 2: sum of anti-*E*. *coli*-LPS + anti-PG-PS, Index 3: sum of anti*-E*. *coli*-LPS + anti-Pg-LPS + anti-PG-PS.

The mTSS values at the first serum collection (shown as median and interquartile range (IQR)), in the RRP group were 37 (14–68), slightly higher than 23 (17–76) in the non-RRP group, but was not statistically significant. However, the median (IQR) of disease duration in RRP group was 48 (23–151) months, and shorter than 116 (52–174) months in the non-RRP group, indicating joint destruction progresses more rapidly in the RRP group than in non-RRP group. As expected, the annual change of mTSS during a 1 year period (ΔmTSS/y) determined at joints of the hands and feet was 5.8 (2.2–11) in the RRP group, and significantly higher than the annual change value of 0.0 (0–0.8) in the non-RRP group (p<0.0001). On the other hand, there was no difference in either the prevalence or antibody level of anti-CCP antibody between the RRP and the non-RRP groups. The median (IQR) of anti-CCP antibody titer was 3.7 (0.8–8.7) units/ml in RRP and 1.1 (0.2–20) units/ml in the non-RRP group compared to 0.08 (0.047–0.099) in NL controls. If sera contained more than 0.1 units/ml of anti-CCP antibody (higher than the 3rd quartile of NL controls), they were considered “positive” for anti-CCP antibody. Accordingly, 51/54 (94%) in the RRP group were positive for anti-CCP antibody, while 87/101 (84%) were positive in the non-RRP group. In addition, the average age of the NL group (42±14 years old) was significantly younger than that of patients with RRP (62±14) and non-RRP (66±11). Despite of these differences, there were no significant differences in IgG and IgA antibody levels against all environmental pathogens and their IgA/IgG antibody ratio between NL, RRP and non-RRP groups as shown in Fig 1.

Firstly, to characterize the antibody responses to environmental pathogens, the possible effects of aging and disease duration on antibody levels were examined. Importantly, no age and no disease duration-associated changes in antibody levels were observed in NL, RRP, and non-RRP groups (S1 Fig and S2 Fig), with one exception. IgG anti-Pg-LPS antibody levels increased with age in NL group (p = 0.0433) (S1 Fig). These observations were confirmed by assaying IgG and IgA antibody levels against these pathogens in sera from 10 patients with RA treated with MTX for 13–113 months. Importantly, IgG and IgA antibody levels against all antigens assayed, except Pg-LPS, remained unchanged, regardless of the long-term treatment with MTX (S3 Fig). Based on these observations, it was considered that the antibody responses to environmental pathogens are unique to individuals, and generally remain at the same levels. Interestingly, apparent increases in IgG and especially IgA antibody responses to Pg-LPS were observed in these patients treated with MTX, indicating a possible adverse effect of MTX. To address this possibility, further detailed studies are required to reveal whether MTX affects oral and/or intestinal bacterial components.

To further characterize the antibody responses to environmental pathogens, the relationship between IgG and IgA antibody responses to individual pathogens was analyzed (S4 Fig). IgG and IgA antibody levels against *E*. *coli*-LPS, Pg-LPS and PG-PS positively correlated (p<0.05) in all groups, indicating that IgG and IgA antibody production is orchestrated depending on the intestinal and oral environment of individuals. Importantly, no correlation was observed between IgG antibody levels against individual pathogens in the NL group. To the contrary, IgG anti-Pg-LPS antibody levels positively correlated with IgG anti-PG-PS antibody levels (p<0.0001) in RRP, whereas IgG anti-*E*. *coli*-LPS antibody levels correlated with IgG anti-PG-PS antibody levels (p = 0.0454) in non-RRP. Similarly, no correlation was observed between IgA antibody levels against individual pathogens in all groups, except IgA anti-Pg-LPS and IgA anti-PG-PS antibody levels correlated well in non-RRP (p<0.0063). These observations indicated potential differences in the bacterial compositions of the digestive system between NL, RRP and non-RRP. Importantly, the positive correlation of IgG antibody responses with certain pairs of pathogens in RA indicate a possibility that patients with low IgG antibody response against one pathogen have lower antibody response to other pathogens. Based on these basic features of antibodies, the comprehensive IgG and IgA antibody responses in individual patients were calculated, and expressed as IgG and IgA index 1–3, respectively. However, as shown in Fig 1B, no difference was observed in either IgG or IgA Indexes between NL and RA in this study employing a limited number of pathogenic agents.

### 2. Linkage of antibody responses to bacterial pathogens with disease markers

To understand the etiology of RA, it is important to consider that the disease process may begin many years before the clinical diagnosis of RA, and arthritis is just one distinctive symptom among multiple symptoms caused by systemic disorders. From this point of view, it is critical to identify the putative pathogens, which evoke disease markers, such as RF, ESR, CRP and anti-CCP antibody and cause hematological abnormality, and ultimately are implicated in developing autoimmune diseases.

**Fig 2:**
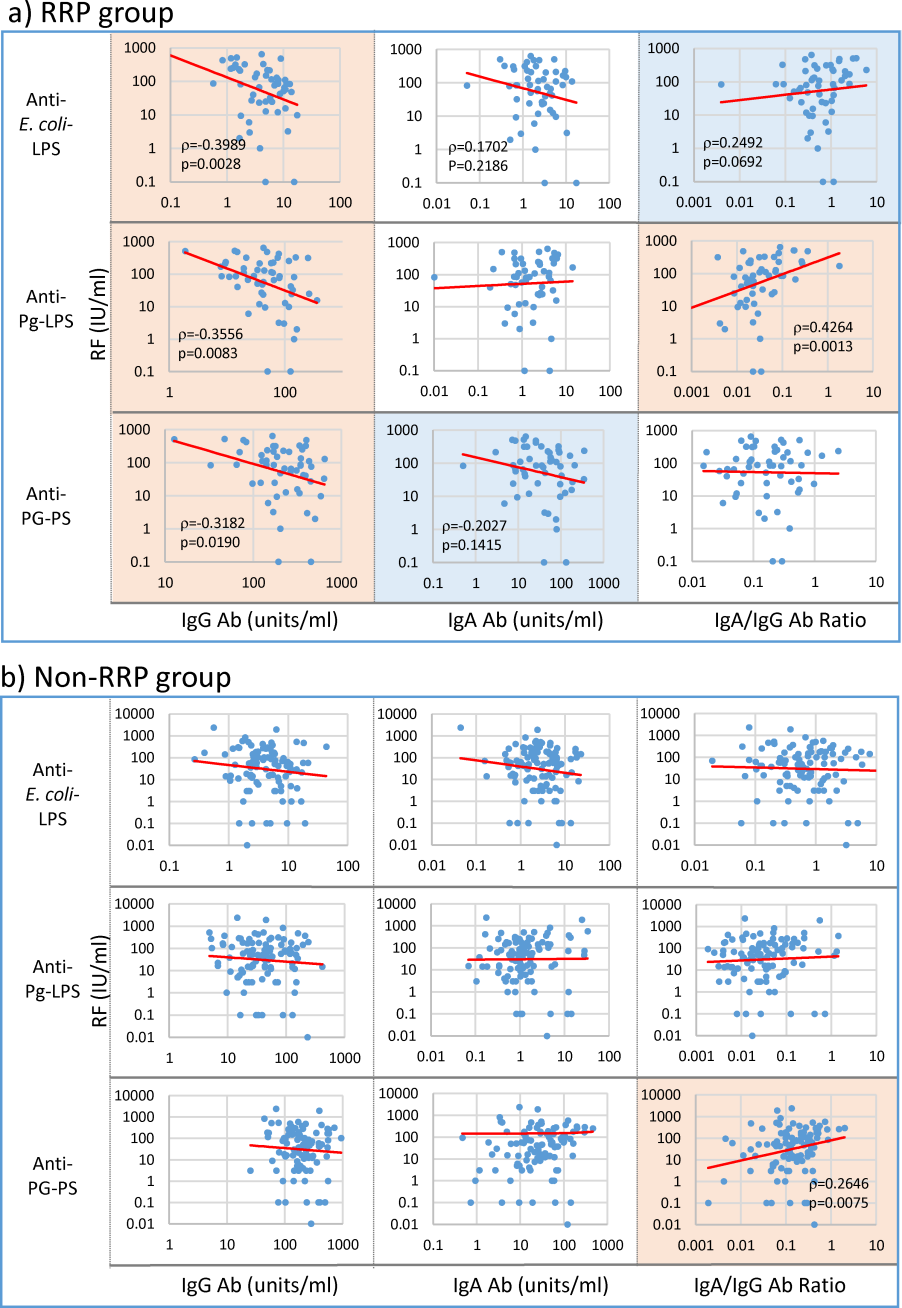
Linkage of RF with IgG and IgA antibody responses to bacterial pathogens in RA patients. IgG and IgA antibody levels against individual pathogens and their IgA/IgG antibody ratio were analyzed for possible correlation with RF levels in 54 patients with RRP (a) and 101 patients with non-RRP (b) by Spearman’s rank correlation coefficient analysis. NOTE: Pink: significant correlation at p<0.05, Blue: trending toward correlation at 0.05≤p<0.15, No color: no correlation.

At first, to clarify a possible involvement of environmental pathogens in RA, the relationship between antibody responses to three bacterial pathogens and serological disease marker levels was analyzed by Spearman’s non-parametric rank correlation analysis. As shown in Fig 2A, RF levels in the RRP group inversely correlated with IgG antibody levels against *E*. *coli*-LPS (p = 0.0028), Pg-LPS (p = 0.0083) and PG-PS (p = 0.0190), and tended to correlate with IgA anti-*E*. *coli*-LPS (p = 0.2186) and anti-PG-PS (p = 0.1415) antibody levels. Consequently, RF levels correlated well with the IgA/IgG anti-Pg-LPS antibody ratio (p = 0.0013) and tended to correlate with the IgA/IgG anti-*E*. *coli*-LPS antibody ratio (p = 0.0692). On the other hand, no apparent relationship between RF and both IgG and IgA antibody levels against any pathogens was observed in the non-RRP group. However, it was notable that RF levels in this group apparently correlated with the IgA/IgG anti-PG-PS antibody ratio (p = 0.0075) as shown in Fig 2B.

**Fig 3:**
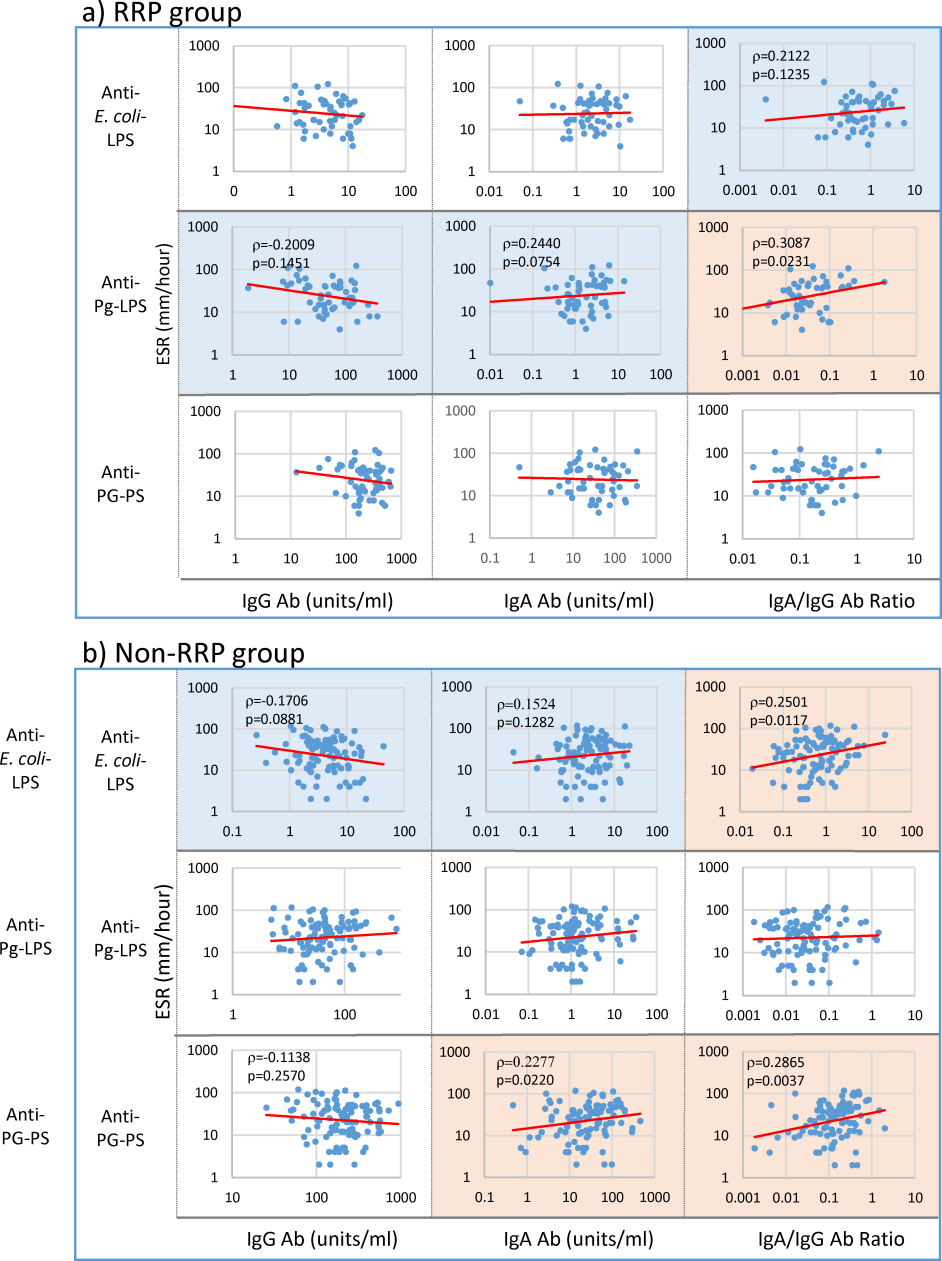
Linkage of ESR with IgG and IgA antibody responses to bacterial pathogens in RA patients. IgG and IgA antibody levels against individual pathogens and their IgA/IgG antibody ratio were compared with ESR in 54 patients with RRP (a) and 101 patients with non-RRP (b) by Spearman’s rank correlation coefficient analysis. NOTE: Pink: significant correlation at p<0.05, Blue: trending toward correlation at 0.05≤p<0.15, No color: no correlation.

Similarly, ESR in the RRP group tended to correlate inversely with IgG (p = 0.1451) and positively with IgA anti-Pg-LPS (p = 0.0754) antibody levels, and consequently correlated well with the IgA/IgG anti-Pg-LPS antibody ratio (p = 0.0231) (Fig 3A). On the other hand, ESR in non-RRP tended to be associated with lower IgG antibody responses to *E*. *coli*-LPS (p = 0.0881) and high IgA antibody responses to *E*. *coli*-LPS (p = 0.1281) and PG-PS (p = 0.0220), and consequently correlated well with the IgA/IgG anti-*E*. *coli*-LPS (p = 0.0117) and anti-PG-PS antibody ratio (p = 0.0037) (Fig 3B).

**Fig 4:**
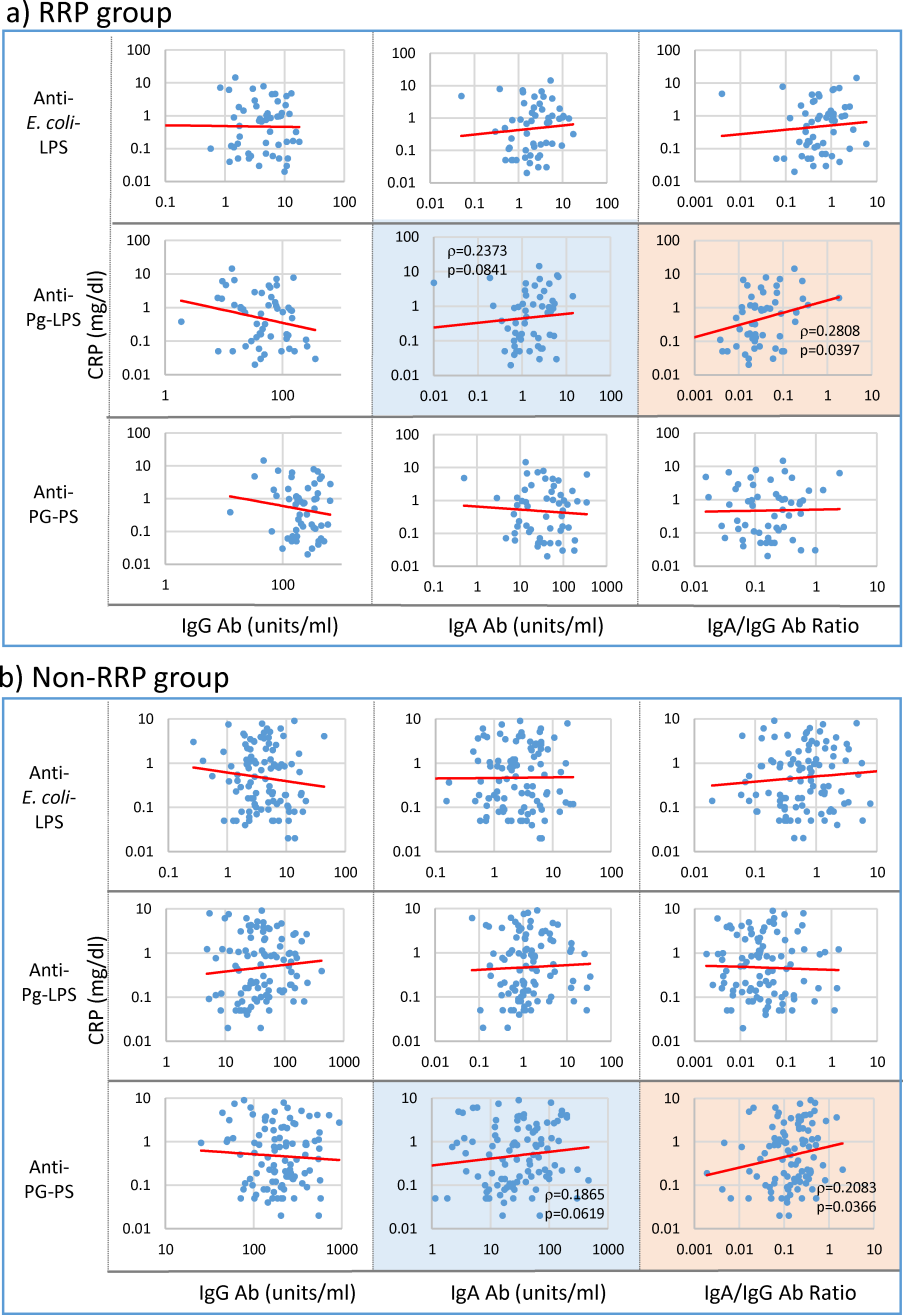
Linkage of CRP with IgG and IgA antibody responses to bacterial pathogens in RA patients. IgG and IgA antibody levels against individual pathogens and their IgA/IgG antibody ratio were compared with CRP levels in 54 patients with RRP (a) and 101 patients with non-RRP (b) by Spearman’s rank correlation coefficient analysis. NOTE: Pink: significant correlation at p<0.05, Blue: trending toward correlation at 0.05≤p<0.15, No color: no correlation.

Like ESR, CRP levels correlated with the IgA/IgG anti-Pg-LPS (p = 0.0397) antibody ratio in RRP (Fig 4A), whereas these correlated with the IgA/IgG anti-PG-PS antibody ratio (p = 0.0366) in non-RRP (Fig 4B).

On the other hand, no apparent correlation was observed between anti-CCP antibody levels and antibody responses to any pathogens in both RRP and non-RRP, although anti-CCP antibody levels tended to inversely correlate with IgG anti-*E*. *coli*-LPS (p = 0.0706), and positively correlate with IgA anti-PG-PS (p = 0.0697) and the IgA/IgG anti-PG-PS ratio (p = 0.0599) in non-RRP (data not shown). These observations indicate that anti-CCP antibody production is not directly influenced by these pathogens tested, and likely to be elicited by a combination of multiple pathogens as discussed later, or by other pathogens such as leukotoxin A as suggested by Konig et al [59].

### 3. Linkage of antibody responses to bacterial pathogens with severity of arthritis

**Fig 5:**
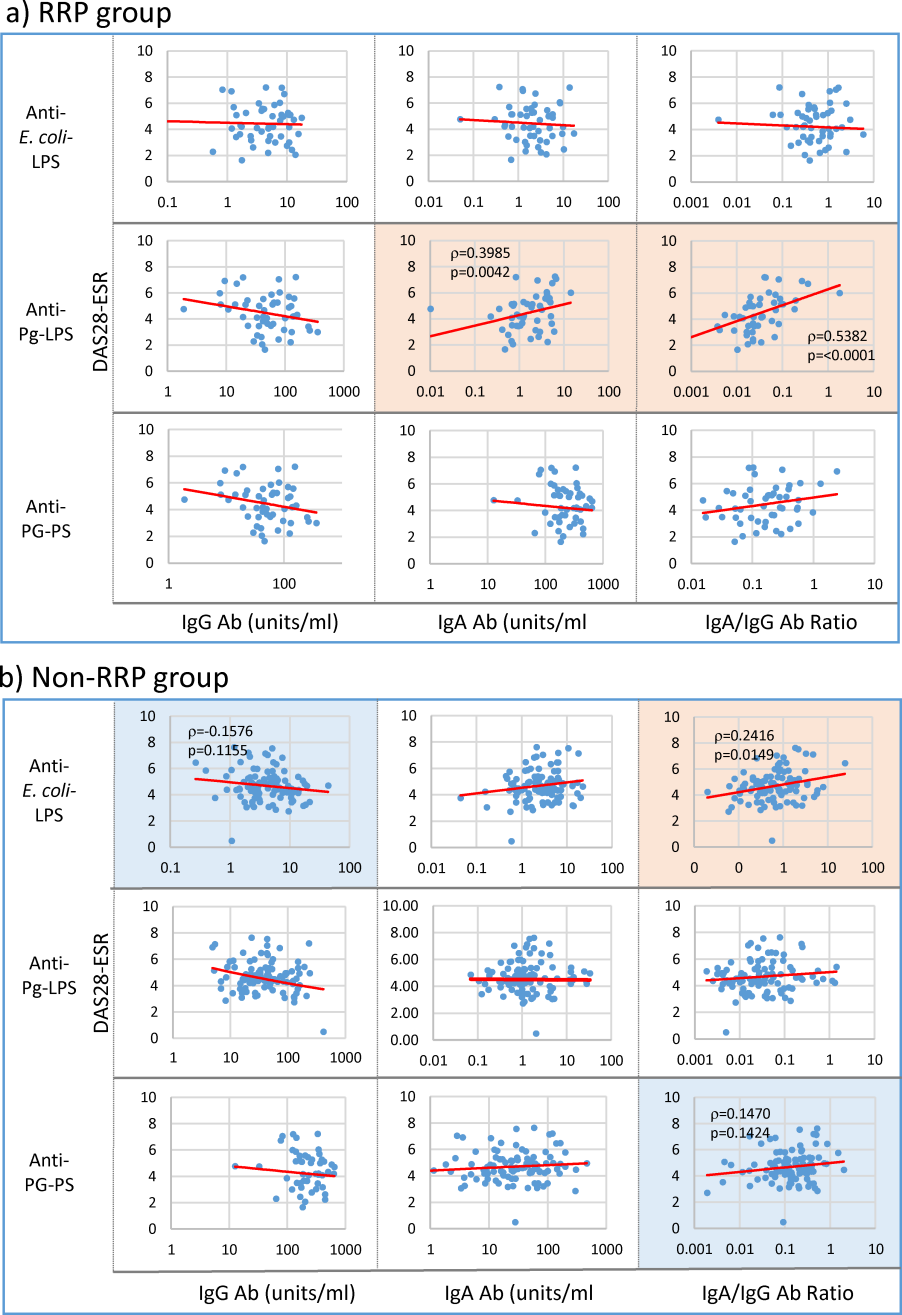
Linkage of DAS28-ESR with IgG and IgA antibody responses to bacterial pathogens in RA patients. IgG and IgA antibody levels against individual pathogens and their IgA/IgG antibody ratio were compared with DAS28-ESR score values in 54 patients with RRP (a) and 101 patients with non-RRP (b), using Spearman’s rank correlation coefficient analysis. NOTE: Pink: significant correlation at p<0.05, Blue: trending toward correlation at 0.05≤p<0.15, No color: no correlation.

The observations described above indicate a possibility that a variety of environmental pathogens, which overwhelm the host’s antibody responses for an extended period of time, may play essential roles in evoking and increasing disease marker levels, and consequently perpetuating and aggravating inflammatory arthritis. To examine this possibility, we analyzed potential association between IgG and IgA antibody levels against individual pathogens and severity of arthritis by Spearman’s non-parametric rank correlation analysis. As shown in Fig 5A, DAS28-ESR values correlated specifically with IgA anti-Pg-LPS antibody levels (p = 0.0042) and the IgA/IgG anti-Pg-LPS antibody ratio (p<0.0001) in the RRP group as well as RF (Fig 2A), ESR (Fig 3A) and CRP (Fig 4A) levels. Importantly, SJC28 and TJC28 correlated with IgA/IgG anti-Pg-LPS ratio (p = 0.0042 and p = 0.0025), and pVAS scores correlated with IgA anti-Pg-LPS antibody levels (p = 0.0198) in the RRP group (data not shown).

On the other hand, DAS28-ESR values in the non-RRP group positively correlated with the IgA/IgG anti-*E*. *coli*-LPS antibody ratio (p = 0.0149), but did not correlate with either anti-Pg-LPS or anti-PG-PS antibodies as shown in Fig 5B. However, pVAS score inversely correlated with IgG anti-Pg-LPS antibody levels (p = 0.0006) and positively correlated with the IgA/IgG anti-Pg-LPS antibody ratio (p = 0.0040) (data not shown), indicating that Pg-LPS also contributes to pathogenesis the non-RRP group. Importantly, no correlation was observed between IgG and IgA anti-PG-PS antibody levels and the severity of arthritis in this group (Fig 5B), even though PG-PS seems to contribute to evoking RF, ESR and CRP (Figs 2B, 3B & 4B). These discrepancies indicate that multiple pathogens, which have unique pathogenic effects, play different pathological roles independently or concomitantly in individual patients with RA, and may affect disease outcomes with distinct disease phenotypes, such as RRP and non-RRP.

### 4. Linkage of serological disease maker levels with severity of arthritis

**Fig 6:**
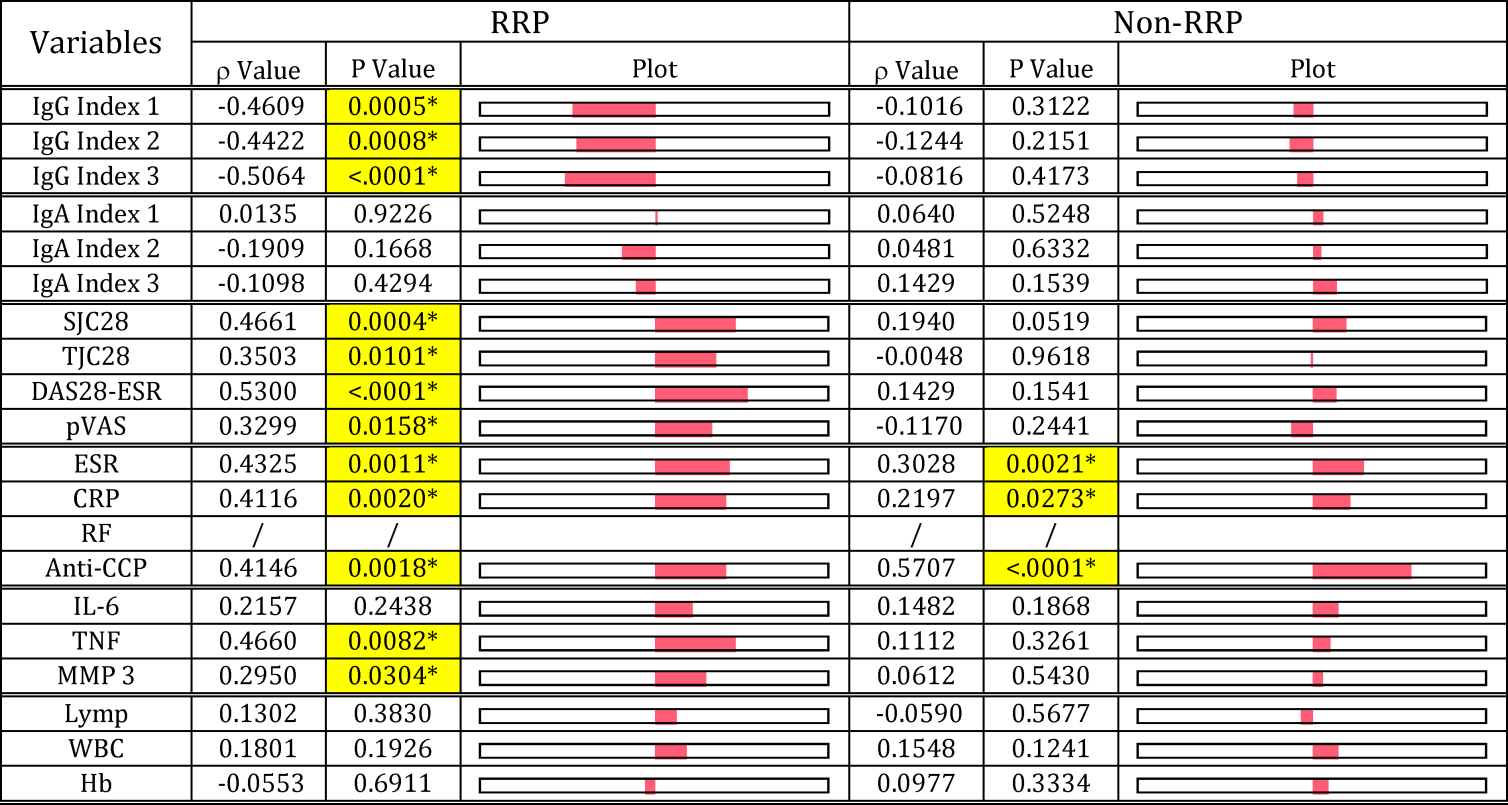
Relationship of RF with antibody response functions and clinical maker levels in patients with RRP and non-RRP. RF levels were compared with IgG and IgA index values, severity of arthritis, disease marker levels, serum cytokine levels, and hematological values in 54 patients with RRP and 101 patients with non-RRP, using Spearman’s rank correlation coefficient analysis. NOTE: Plot: Visual display for positive and negative “ρ” value of Spearmen correlation coefficient. Cells highlighted with yellow indicate significant correlation at p<0.05. Index 1: sum of anti-*E*. *coli*-LPS + anti-Pg-LPS, Index 2: sum of anti-*E*. *coli*-LPS + anti-PG-PS, Index 3: sum of anti*-E*. *coli*-LPS + anti-Pg-LPS + anti-PG-PS.

**Fig 7:**
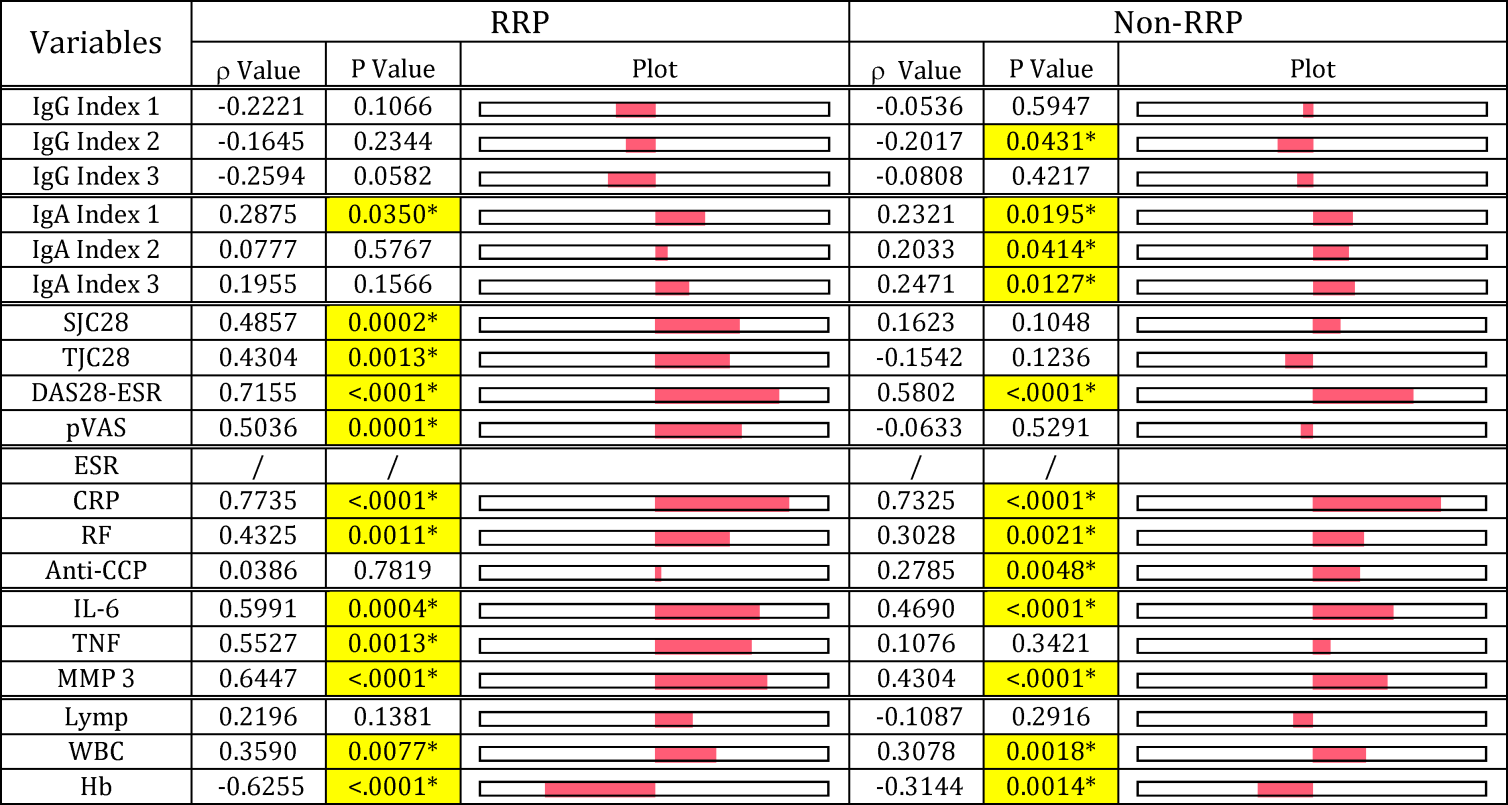
Relationship of ESR with antibody response functions and clinical marker levels in patients with RRP and non-RRP. ESR values were compared with IgG and IgA index values, severity of arthritis, disease marker levels, serum cytokine levels, and hematological values in 54 patients with RRP and 101 patients with non-RRP, using Spearman’s rank correlation coefficient analysis. NOTE: Plot: Visual display for positive and negative “ρ” value of Spearmen correlation coefficient. Cells highlighted with yellow indicate significant correlation at p<0.05. Index 1: sum of anti-*E*. *coli*-LPS + anti-Pg-LPS, Index 2: sum of anti-*E*. *coli*-LPS + anti-PG-PS, Index 3: sum of anti*-E*. *coli*-LPS + anti-Pg-LPS + anti-PG-PS.

**Fig 8:**
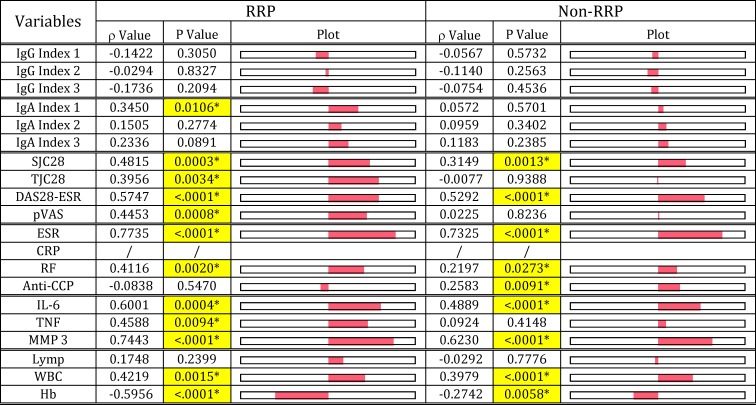
Relationship of CRP with antibody response functions and clinical marker levels in patients with RRP and non-RRP. CRP levels were compared with IgG and IgA index values, severity of arthritis, disease marker levels, serum cytokine levels, and hematological analytical values in 54 patients with RRP and 101 patients with non-RRP, using Spearman’s rank correlation coefficient analysis. NOTE: Plot: Visual display for positive and negative “ρ” value of Spearmen correlation coefficient. Cells highlighted with yellow indicate significant correlation at p<0.05. Index 1: sum of anti-*E*. *coli*-LPS + anti-Pg-LPS, Index 2: sum of anti-*E*. *coli*-LPS + anti-PG-PS, Index 3: sum of anti*-E*. *coli*-LPS + anti-Pg-LPS + anti-PG-PS.

**Fig 9:**
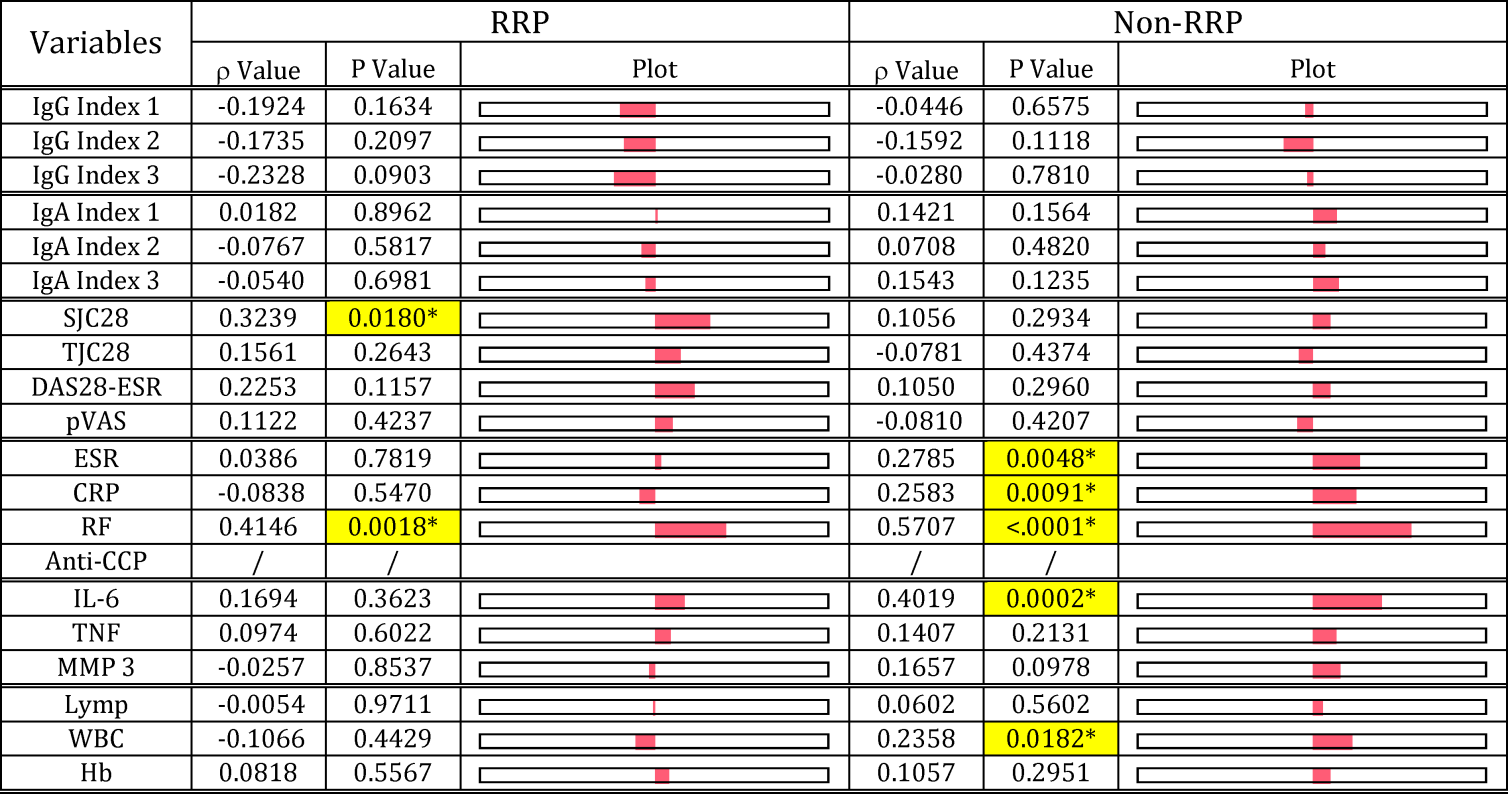
Relationship of anti-CCP antibody levels with antibody response functions and clinical marker levels in patients with RRP and non-RRP. Anti-CPP antibody levels were compared with IgG and IgA index values, severity of arthritis, disease marker levels, serum cytokine levels, and hematological analytical values in 54 patients with RRP and 101 patients with non-RRP, using Spearman’s rank correlation coefficient analysis. NOTE: Plot: Visual display for positive and negative “ρ” value of Spearmen correlation coefficient. Cells highlighted with yellow indicate significant correlation at p<0.05. Index 1: sum of anti-*E*. *coli*-LPS + anti-Pg-LPS, Index 2: sum of anti-*E*. *coli*-LPS + anti-PG-PS, Index 3: sum of anti*-E*. *coli*-LPS + anti-Pg-LPS + anti-PG-PS.

To obtain a clear picture of the etiopathogenesis of RA, we investigated the relationship of serological disease marker (RF, ERS, CRP and anti-CCP antibody) levels with arthritis severity (SJC28, TJC28, DAS28-ESR and pVAS), and hematological maker (lymphocytes, WBC and Hb) levels (Figs 6–9). In the RRP group, RF levels, which inversely correlated with IgG anti-*E*. *coli*-LPS, Pg-LPS and PG-PS, inversely correlated with IgG index 1–3 values as expected (Fig 6). Importantly, RF levels correlated well with severity of arthritis (SJC28, TJC28, DAS28-ESR and pVAS), serological disease maker (ESR, CRP, anti-CCP antibody) and inflammatory marker (TNF, MMP3) levels in RRP as shown in Fig 6. These observations indicate that pathogens evoking RF may directly contribute to triggering and exacerbating inflammatory reaction in the RRP group. On the other hand, RF levels in the non-RRP group did not correlate with either IgG or IgA index values, and furthermore either arthritis or inflammatory marker levels, although RF levels correlated well with ESR, CRP, and anti-CCP antibody levels (Fig 6). These data indicate that different pathogens or different combinations of pathogens are involved in evoking RF in the RRP and non-RRP groups. Notably, RF levels correlated well with anti-CCP antibody levels in both RRP and non-RRP groups, regardless of the significant differences in the profiles of RF and the progression of arthritis between the two groups. This indicates that RF may trigger CCPs and subsequent anti-CCP antibody production, regardless of the phenotype of disease, as suggested by Carmona-Rivera et. al. [60].

On the other hand, ESR tended to correlate with IgA index values rather than IgG index values in both RRP and non-RRP (Fig 7). Importantly, like RF, ESR correlates with arthritis (SJC28, TJC28, DAS28-ESR and pVAS) scores and inflammatory maker (TNF and MMP-3) levels in the RRP group as shown in Fig 7, indicating that Pg-LPS, which contributes to increasing ESR (Fig 3), may also be implicated in developing arthritis in the RRP group. Similarly, ESR in the non-RRP group also correlated well with DAS28-ESR, but did not correlate with other arthritis marker levels in this group. This indicates that *E*. *coli*-LPS, PG-PS or both, rather than Pg-LPS, are implicated in the severity of arthritis in the non-RRP group. Due to the possible difference in the pathogens involved in evoking ESR in the RRP and non-RRP groups, a significant difference was observed in the ESR profiles between the RRP and non-RRP groups; ESR did not correlate with anti-CCP antibody levels (p = 0.7819) in the RRP group, but correlated well (p = 0.0048) in the non-RRP group. Importantly, ESR correlated well with WBC in both RRP and non-RRP, indicating that the pathogens which evoke ESR may contribute to acute inflammatory reaction regardless of RA phenotype (Fig 7).

Similar patterns to those observed in ESR were observed in the relationship between CRP levels and arthritis scores and inflammatory marker levels, including WBC and Hb in both RRP and non-RRP groups (Fig 8). This indicates that the pathogens that increase ESR also contribute to increasing CRP levels in RA.

Furthermore, Hb levels were inversely correlated with ESR and CRP levels, but not with RF levels in both RRP and non-RRP groups (Figs 7 & 8), indicating pathogens that evoke ESR and CRP exert an adverse effect on Hb levels in RA. In fact, Hb levels strongly correlated with IgA anti-*E*. *coli*-LPS antibody levels (P = 0.0005) and IgA/IgG anti-*E*. *coli* antibody ratio (p = 0.0181) in the non-RRP group (data not shown). On the other hand, no correlation was observed between Hb levels and either IgG or IgA antibody responses to any pathogens in the RRP group. However, it is likely that Pg-LPS alone or in combination with *E*. *coli*-LPS may contribute to reducing Hb levels in the RRP group. This is evidenced by the fact that Hb levels were tightly correlated with ESR and CRP levels, which are linked to low IgG and high IgA antibody responses to Pg-LPS in this group.

Unlike these classic disease makers, anti-CCP antibody levels, which did not correlate with antibody responses to any pathogens, did not correlate with either IgG or IgA Index values in both RRP and non-RRP groups, but correlated well with RF levels in both groups (Fig 9). Although anti-CCP antibody levels did not correlate with DAS28-ESR and inflammatory marker levels (IL-6, TNF and MMP-3) unlike RF, ESR and CRP, but correlated with SJC28 (p = 0.0180) in the RRP group, and IL-6 (p = 0.0002) in the non-RRP group.

**Fig 10:**
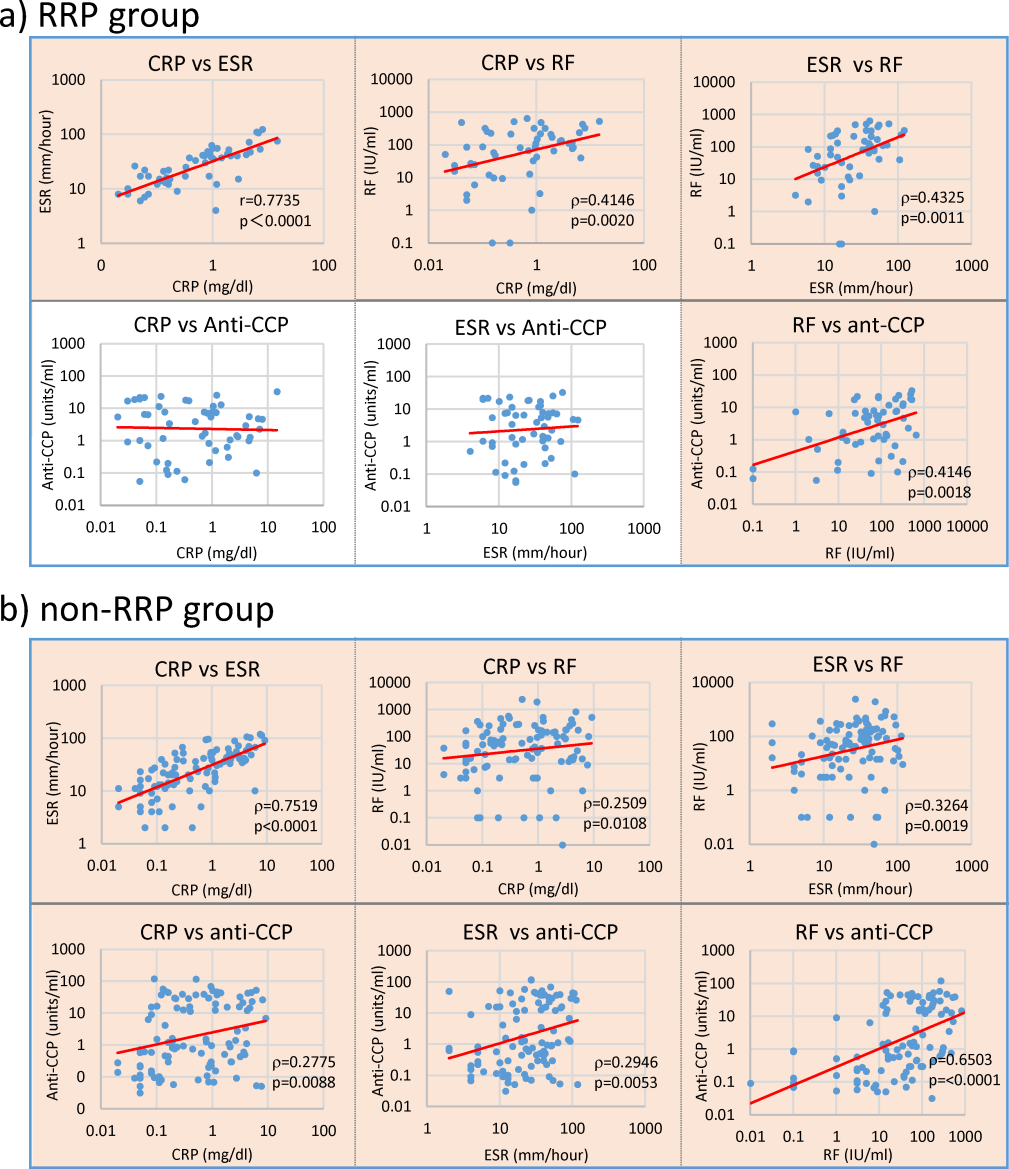
Differences in the relationships between individual disease markers in the RRP and non-RRP groups. Potential correlations between individual disease marker levels in 54 patients with RRP and 101 patients with non-RRP were confirmed by Spearman’s rank correlation coefficient analysis. NOTE: Pink: significant correlation at p<0.01, No color: no correlation.

These data indicate that anti-CCP antibodies may not play a dominant arthritogenic role, but are implicated in joint tissue damage at some stage of the inflammatory process. A remarkable difference between the RRP and non-RRP groups was that anti-CCP antibody levels did not correlate with CRP and ESR levels in RRP (Fig 10A), but closely correlated with these in the non-RRP group as shown Fig 10B. These differences indicate that bacterial pathogenic components such as Pg-LPS, which are assumed to dominantly contribute to increasing CRP and ESR in the RRP group, are less likely to be involved in eliciting anti-CCP antibody in the RRP group. On the other hand, *E*. *coli*-LPS, PG-PS or both combined, which affect CRP and ESR levels in the non-RRP group, may contribute to eliciting anti-CCP antibody by enhancing neutrophil extracellular traps (NETs) formation or activating synovial cells [60] as discussed later.

**Fig 11:**
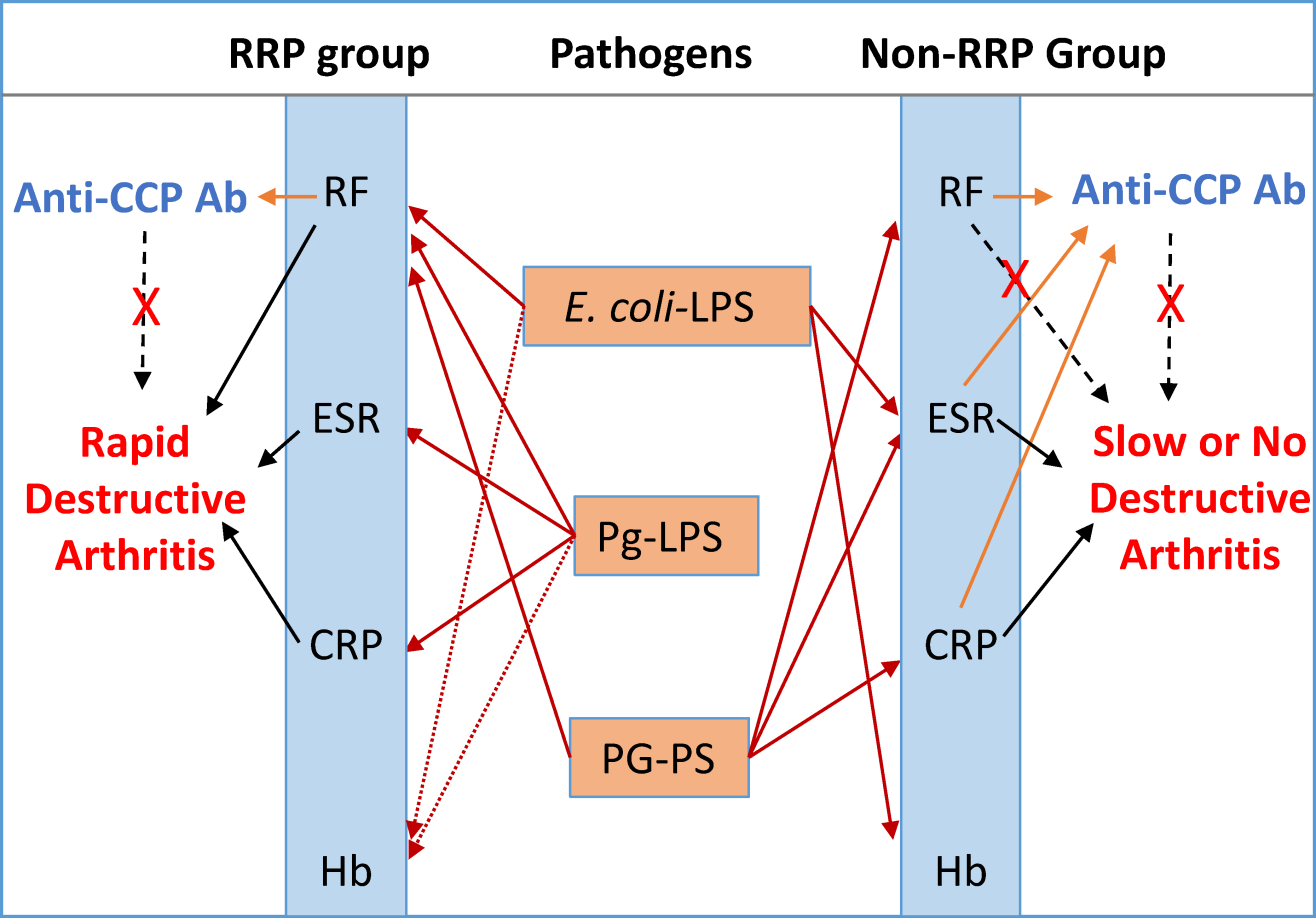
Multiple bacterial pathogens are implicated in evoking serological disease markers and consequently aggravating disease activity in the RRP and non-RRP groups. Low IgG antibody responses to *E*. *coli*-LPS, Pg-LPS and PG-PS are linked to RF, ESR and CRP levels in RRP. Among these putative pathogens, Pg-LPS and related pathogens derived from oral bacteria may play critical pathological roles in the RRP group. On the other hand, *E*. *coli*-LPS, PG-PS and other related pathogenic components, instead of Pg-LPS, may contribute to increasing RF, ESR and CRP levels in non-RRP. Anti-CCP antibody levels clearly correlate with RF in both RRP and non-RRP groups, but are not linked to severity of arthritis. NOTE: Arrows with solid line: Linkage, Arrows with dotted line: Possible linkage, Dashed line with X: No apparent linkage.

Taken together, we concluded that various types of bacterial pathogens, which exert unique pathological effects, may be actively involved in evoking and aggravating disease maker levels and disease activity independently and collectively, and play critical roles in the pathogenesis of RA as summarized in Fig 11.

## Discussion

The most important finding in this study is that IgG antibody responses to environmental pathogens inversely correlate or tend to correlate with serological disease marker levels and severity of arthritis, whereas IgA antibody levels and the IgA/IgG antibody ratio positively correlate or tend to correlate with those marker levels among the patients with RA despite no apparent defect in the antibody response function as shown in Fig 1. More specifically, IgG anti-*E*. *coli*-LPS, Pg-LPS and PG-PS antibody levels inversely correlated with RF levels, whereas the IgA/IgG anti-Pg-LPS antibody ratio positively correlated not only with RF levels, but also ESR, CRP and DAS28-ESR in the RRP group (Figs 2A, 3A, 4A and 5A). In contrast, no apparent relationship was observed between IgG antibody levels and either serological disease marker levels or severity of arthritis in the non-RRP group, but IgA anti-*E*. *coli*-LPS and PG-PS antibody levels and the IgA/IgG anti-*E*. *coli*-LPS and anti-PG-PS antibody ratios positively correlated or tended to correlate with those marker levels (Figs 2B, 3B, 4B and 5B).

One of the unique features of the mucosal immune system is the production of a large amount of non-specific poly-reactive IgA and antigen-specific IgA antibodies, which prevent the penetration or translocation of pathogenic substances such as bacteria and their components into the lamina propria and the blood stream [61, 62]. On the other hand, antigen-specific IgG is produced by B-cells stimulated by antigens escaped from the IgA barrier, and may play a critical role in protecting the host from pathogens that enter the body. Therefore, we assume that the correlation between IgA/IgG antibody ratio and disease activity in RA, in spite of no apparent defect in either IgA or IgG antibody responses to the pathogens, indicates that RA patients are exposed to excessive amounts of pathogens, which overwhelm IgA and IgG antibody defense functions.

Taken together, we speculate that a fundamental disorder in RA may be dysbiosis in the intestinal tract and oral cavity or dysfunction of the gastrointestinal mucosal barrier system rather than abnormality in immune function. However, our current findings do not prove that individual pathogens tested in this study actually play critical pathological roles in RA. For example, there is no evidence yet that Pg-LPS actually evokes RF, ESR and CRP, and exacerbates arthritis in the RRP group. In this aspect, it is also important to take into consideration that antibody levels to individual pathogens may reflect the immune defense function not only to the pathogens tested, but also represent the overall immune defense function against related pathogens. We speculate that anti-Pg-LPS antibody levels may reflect the immune defense function not only to Pg-LPS, but also to *P*. *gigivalis* and other periodontal bacteria, and their pathogenic components. Similarly, anti-*E*. *coli*-LPS antibody reponse reflects immune defense function against not only *E*. *coli*-LPS, but also to *E*. *coli* and other intestinal bacteria, and thier pathogenic components. Therefore, futher studies are required to clarify the pathogenic effects of individual pathogens and their combinations in conjunction with studies on dysbiosis of the intestinal tract and oral cavity.

Regarding the etiological signifcance of RF, an apparent difference was observed between the RRP and non-RRP groups. RF levels correlate well with other serological disease markers, arthritis score, and inflammatory marker levels in the RRP group, but not in the non-RRP group (Fig 6), indicating possible differences in the types of pathogens dominantly involved in evoking RF in RRP and non-RRP groups as shown in Fig 2. It was reported that RF production is triggered by serum immune complexes and antigen-primed T-cells [63]. However, our data indicate that a vareity of bacterial pathogens, such as *E*. *coli*-LPS, Pg-LPS and PG-PS, may directly contribute to evoking RF in RA as well as luekotoxin A produced by *A*. *actinomycetemcomitans* [59]. An additional intriguing finding is that ESR and CRP values correlate well not only with severity of arthritis and inflammatory marker levels, but also with Hb levels in both RRP and non-RRP groups (Figs 7 & 8). This evidence indicates that the pathogens that contribute to evoking ESR and CRP have an adverse influence on Hb levels, whereas the pathogens that evoke RF do not. These results again indicate that multiple pathogens with different pathological effects are involved independently and concomitantly in the pathogeneisis of RA. Consequently, the clinical phenotype will vary depending on the dominant pathogens and the combinations of pathogens as shown in Fig 11.

In contrast to these classic serological disease markers, the significance of anti-CCP antibody in the pathogenesis of RA remains unknown, since anti-CCP antibody levels do not correlate either with antibody levels against any pathogens tested or severity of arthritis in both RRP and non-RRP groups (Fig 9). Based on the assumption that RA is linked to periodontitis, it is widely believed that *P*. *gingivalis* peptidylarginine deiminase (PAD) is implicated in the autoimmunity of RA by creating mimic antigen, CCP, by autocitrulination [64–66]. Contrary to the *P*. *gingivalis* PAD hypothesis, Konig et al. [67] reported that *A*. *actinomycetemcomitans*, which releases leukotoxin A, mediates CCP production, but *P*. *gingivalis* and other periodontal pathogens do not. Furthermore, Carmona-Rivera et. al. [60] paid great attention to NETs formed by stimulation with IgM-RF stimulation [68], the source of CCP antigens, which are then internalized by RA synovial fibroblast-like synoviocytes. These synoviocytes act as antigen-presenting cells, which lead to antibody production against CCPs. This observation that RF is involved in eliciting anti-CCP antibodies is rational, and would explain the tight correlation between RF levels and anti-CCP antibody levels in RA (Figs 9 & 10). Importantly, the antigen internalization activity is observed in synoviocytes from RA patients and dermal fibroblasts from psoriasis patients, but not in normal dermal fibroblasts. This indicates that RA synovial cells already display a pro-inflammatory phenotype under the influence of disease-causative pathogens. Therefore, we consider CCPs and subsequent anti-CCP antibody production is not a primary event, which is implicated in the severity and progression of arthritis.

In the clinical field, it is considered that patients positive for IgM-RF or anti-CCP antibodies are at risk for developing RRP [69], which is observed in 10–40% of RA patients despite immediate treatment with MTX [46–48]. Since RRP in the first year of early RA was reported to be a predictive indicator for further disability and joint damage progression [70], it is important to determine whether periodontal pathogenic bacteria contribute to the progression of arthritis or not. Contrary to our expectations, our data indicate that anti-CCP antibody positivity does not reflect either disease activity or prognosis of RA. However, anti-CCP antibody seems not to be a simple hallmark of exposure to *A*. *actinomycetemcomitans* or other periodontal bacteria in patients with RA, because of the significant differences in the anti-CCP antibody profiles between the RRP and non-RRP groups. For example, anti-CCP antibody levels do not correlate with ESR and CRP levels in the RRP group, but correlate well with these in the non-RRP groups (Figs 9 & 10), despite good correlation with RF levels in both groups. These differences between RRP and non-RRP can be explained by the difference in the types of pathogens dominantly involved in evoking RF, which may subsequently elicit anti-CCP antibodies. For instance, Pg-LPS may be the dominant pathogen that contributes to evoking RF in the RRP group, while PG-PS might be the main contributor in the non-RRP group.

These observations indicate that the patients in the RRP group are under the influence of oral pathogenic bacteria or their pathogenic components, such as Pg-LPS produced by *P*. *gingivalis*, leukotoxin-A produced by *A*. *actinomycetemcomitans*, or other related pathogens. On the other hand, patients in the non-RRP group might be affected by intestinal bacteria or their pathogenic components such as *E*. *coli*-LPS and PG-PS. In this regard, it is important to take into consideration that IgG and especially IgA antibody responses to Pg-LPS are significantly increased by prolonged treatment with MTX (S4 Fig). This indicates that MTX may enhance the growth of oral pathogenic bacteria or may disturb the homeostasis of oral and intestinal microbiota composition. Indeed, recent studies indicate the potential of an oral chronic infection to alter arthritis progression in susceptible patients, although the pathological mechanisms involved are not clearly understood. At this point, Nakajima et al. [71] have reported critical findings to explain the possible pathogenic roles of periodontal bacteria. A single oral administration of *P*. *gingivalis* in mice induced significant diversity of microbial communities in the gut, which coincided with the dissemination of enterobacteria to the liver, and decreased mRNA expression of tight junction proteins, leading to an increase in serum endotoxin levels. Importantly, the alteration of gut microbiota composition was not caused by the growth of *P*. *gingivalis* in the intestine, but daily influx of *P*. *gingivalis* to the intestinal lumen affects not only intestinal bacterial flora, but also affects immune defense function at the intestinal mucosa, and increases susceptibility to various types of bacteria and their toxins. Furthermore, Van der Post et al. [72] reported that *P*. *gingivalis*, which is abundant in the oral cavity, but is also found in the colon, produces a proteinase, Arg-gingipain B (RgpB), which is capable of cleaving the mucin layer that protects the colonic epithelial surfaces from the penetration of commensal bacteria, and may play a key role in triggering colitis. We believe that these proposals are a rational way to explain the potential risk of periodontitis for a certain subset of RA patients. Since periodontitis is not specific to RA, and widely distributed even in the general population, we assume that *P*. *gingivalis* influxed into gastrointestinal tract may play critical pathological roles in susceptible patients. In this regard, we consider that immune defense function in the gastrointestinal tract may differ among the NL, RRP and non-RRP groups. For example, it is highly likely that periodontal bacteria and their components may contribute to dysbiosis development or to damage of the mucosal barrier system in the gastrointestinal tract of a certain subset of RA patients such as those with RRP. Therefore, we believe it is important to consider the possible implication of oral pathogenic bacteria in augmentation of disease activity in RA regardless of whether patients have infectious periodontitis or not.

In fact, imbalance of intestinal bacteria was considered as a possible factor in the etiopathogenesis of RA [73, 74]. To study the possible contribution of dysbiotic gut microbiota to the pathogenesis of RA, we treated patients with RA with a natural milk antibody preparation, which contains high levels of antibodies against pathogenic entromicrobes and their toxins. We found that supplemental treatment with this milk antibody preparation effectively reduced arthritis symptoms and improved intestinal disorders in a certain subset of RA patients [75]. Recent studies more clearly indicate dysbiosis in RA as reviewed [27]. More importantly, recent studies show a genetic influence on the composition of the dominant eubacterial population in mice [76] and children [77, 78]. In addition, Gomez et. al. [79] reported that the intestinal bacterial population of CIA-susceptible DRβ1 0401 transgenic mice is dominated by a *Clostridium-*like bacterium, whereas the guts of CIA-resistant 0402 transgenic mice are enriched for members of the *Porphyromonadaceae* family and *Bifidobacteria*. Furthermore, Totaro et al. [80] reported that patients with undifferentiated peripheral inflammatory arthritis and RA, who carry the HLA-DRB1*04 allele, showed a high positivity for *P*. *gingivalis* DNA in the synovial tissues compared to patients negative for the allele. These observations indicate that oral and intestinal bacteria composition is under the influence of HLA types, and the gut microbiome will be a potential biomarker for susceptibility to arthritis.

Importantly, regardless of being pathogenic or non-pathogenic, bacteria commonly carry pathogen-associated molecular patterns on their surfaces, which are recognized by pattern recognition receptors (PRRs) such as toll-like receptor (TLRs), and may activate host innate and adaptive immune systems. Therefore, it is highly likely that excess amounts of a variety of potential pathogens, which overwhelm the host immune defense functions, stimulate the PRRs, and trigger an uncontrollable inflammatory reaction, leading to the development of autoimmune diseases under certain conditions [22, 81, 82]. In this study, we tested antibody responses to three potential pathogens in patients with RA, and concluded that a variety of commensal and pathogenic microbes and their components may play pathological roles under certain conditions. Lastly, we believe that oral pathogenic bacteria such as *P*. *gingivalis* and others influxed into the intestinal tract might play indirect but critical pathogenic roles in enhancing and perpetuating inflammatory synovitis and osteitis, leading to severe joint damage in a subset of RA patients such as classified into the RRP group, regardless these patients are affected by periodontal infection or not.

## Conclusion

A variety of bacterial pathogens overwhelming host defense function may play critical pathogenic roles independently, collectively, and/or synergistically, and contribute to evoking serological disease marker levels and aggravating disease activity in RA. The outcomes of disease vary significantly depending on the types and combinations of pathogens dominantly involved. This concept will be applied for studying the pathogenesis of other autoimmune diseases.

## Supporting information

S1 Appendix “An ELISA protocol to improve the accuracy and reliability of serological antibody assays”. (PDF)

S1 Fig Effect of aging on IgG and IgA antibody responses to bacterial pathogens. IgG and IgA antibody levels against *E*. *coli*-LPS, Pg-LPS and PG-PS were determined in sera from 38 NL controls, 54 patients with RRP and 101 patients with non-RRP, and plotted against age. Antibody levels against these pathogens were not affected by age in both NL and RA groups, except IgG anti-Pg-LPS antibody, which increased with age in the NL controls, whereas tended to decrease in the RRP group. NOTE: Pink: significant correlation at p<0.05, Blue: trending toward correlation at 0.05≦p<0.15, No color: no correlation.
(TIF)

S2 Fig Effect of disease duration on IgG and IgA antibody responses to bacterial pathogens. IgG and IgA antibody levels against *E*. *coli*-LPS, Pg-LPS and PG-PS were determined in sera from 54 patients with RRP and 101 patients with non-RRP, and plotted against disease duration (months). No apparent antibody level change associated with disease duration was observed. This evidence was confirmed in a separate study on the effect of therapeutics on IgG and IgA antibody responses in patients with RA.
(TIF)

S3 Fig Effect of MTX on IgG and IgA antibody responses to potential pathogens. IgG and IgA antibody levels against *E*. *coli*-LPS, Pg-LPS and PG-PS were determined in sera collected multiple times from 7 patients with RRP and 3 patients with non-RRP, who were treated with MTX for 13 to 113 months. MTX treatment did not affect anti-*E*. *coli*-LPS and PG-PS, but apparently increased IgG and IgA antibody responses to Pg-LPS. NOTE: dotted black line: RRP, dotted red line: non-RRP, solid black line: an average ± SD at 20 months. Pink: significant correlation at p<0.05. No color: no correlation.
(TIF)

S4 Fig Characterization of IgG and IgA antibody responses to pathogens in NL and RA. IgG and IgA antibody levels against *E*. *coli*-LPS, Pg-LPS and PG-PS were determined in sera from 38 NL controls, 54 patients with RRP and 101 patients with non-RRP, and analyzed potential correlation between antibody responses against individual pathogens by Spearman non-parametric rank correlation analysis. IgG antibody levels against individual pathogens correlated or tended to correlate with IgA antibody levels in both NL and RA. By contrast, IgG antibody levels to individual pathogens did not correlate with IgG antibody levels against other pathogens in NL controls, indicating that IgG antibody responses to individual pathogens are unrelated independent event. However, IgG anti-Pg-LPS antibody levels correlated with IgG anti-PG-PS antibody levels in RRP, and IgG anti-*E*. *coli*-LPS antibody levels correlated with IgG anti-PG-PS antibody levels in non-RRP, indicating antibody responses to Pg-LPS and PG-PS in RRP and antibody responses to *E*. *coli*-LPS and PG-PS in non-RRP are orchestrated.
NOTE: Plot: Visual display for positive and negative “ρ” value of Spearmen correlation coefficient. Cells highlighted with yellow indicate significant correlation at p<0.05, and blue indicate a trend at p<0.0.1.
(TIF)

S1 File Study protocol. (PDF)

S2 File Study protocol–English. (DOCX)
