# Supplementary material for: Contribution of bacterial pathogens to evoking serological disease markers and aggravating disease activity in rheumatoid arthritis
Source: PLoS One. 2018 Feb 6;13(2):e0190588. doi: 10.1371/journal.pone.0190588 (PMC5800560; doi:10.1371/journal.pone.0190588)
Supplement: S1 Appendix — (PDF) [file pone.0190588.s001.pdf]

MethodsX 4 (2017) 153–165

Contents lists available at ScienceDirect

MethodsX

journal homepage: www.elsevier.com/locate/mex

Protocol Article

An ELISA protocol to improve the accuracy and reliability of serological antibody assays

Takaki Waritani, Jessica Chang, Bonnie McKinney, Kuniaki Terato*

Chondrex Inc., 2607 151st NE, Redmond, WA 98052, United States of America

GRAPHICAL ABSTRACT

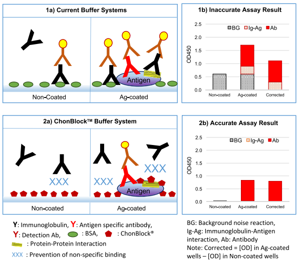

ABSTRACT

To assay serum antibodies by indirect ELISA, it is critical to eliminate a variety of false positive and negative reactions attributed to the principle. These include 1) the background (BG) noise reaction caused by hydrophobic binding of immunoglobulin components in sample specimens to solid surfaces, 2) false positive reaction caused by non-specific binding of immunoglobulins to target-antigens by protein-protein interactions, and 3) other false positive and negative reactions caused by buffer components. No current blocking agents can prevent these false positive and negative reactions, and antibody assay results vary significantly depending on the buffer system used. To address these fundamental problems, we investigated all types of non-specific reactions involved in indirect ELISAs, and the blocking efficacy of current buffer systems and a newly developed ELISA buffer, ChonBlock™. The accuracy and reliability of these assay results were examined in detail by inhibition tests in individual buffer systems. Based on these studies, we are providing a definitive ELISA protocol for all users to improve ELISA technique and obtain accurate, reliable, and reproducible assay data against a variety of antigens.

Abbreviations: ELISA, enzyme-linked immunosorbent assay; BL, blank; BG, background; OD, optical density; NGS, normal goat serum; RA, rheumatoid arthritis; NL, normal; CCP, cyclic citrullinated peptide; LPS, lipopolysaccharide.

* Corresponding author.
E-mail addresses: twaritani@chondrex.com (T. Waritani), jchang@chondrex.com (J. Chang), bmckinney@chondrex.com (B. McKinney), terato@chondrex.com (K. Terato).

http://dx.doi.org/10.1016/j.mex.2017.03.002
2215-0161/© 2017 The Author(s). Published by Elsevier B.V. This is an open access article under the CC BY license (http://creativecommons.org/licenses/by/4.0/).

© 2017 The Author(s). Published by Elsevier B.V. This is an open access article under the CC BY license (http://creativecommons.org/licenses/by/4.0/).

ARTICLE INFO
Article history: Received 19 December 2016; Accepted 23 March 2017; Available online 30 March 2017

Description of protocol

The enzyme-linked immunosorbent assay (ELISA) system is widely used to assay antibodies and antigens without fully comprehending the numerous vexing phenomena attributed to the principle, which utilizes the high binding affinity of proteins to solid surfaces such as micro-titer plates and latex beads. In the indirect ELISA system for serological antibody assays, the inherent high binding affinity of serum immunoglobulins to solid surfaces creates strong false positive BG noise reaction. Unfortunately, this BG noise reaction is not taken into consideration and not determined as a negative control in antigen non-coated wells. Therefore, data influenced largely by this BG noise reaction [1] has led to numerous uncertain conclusions and misunderstandings as discussed [2–4]. To prevent further misuse of the ELISA technique and misinterpretation of serological antibody assay data, it is important to reconsider the principle of the immunoassay system and all types of non-specific reactions involved [5]. The following are basic issues that all ELISA users must take into consideration before setting up an ELISA system for assaying antibodies.

Non-specific reactions involved in indirect ELISA

Background (BG) noise reaction caused by serum samples

In an indirect ELISA for antibody assays, various types of false positive and negative reactions are involved, regardless of antigens. Of these non-specific reactions, the most intense false positive reaction is BG noise reaction caused by hydrophobic binding of immunoglobulin components in sample specimens to solid surfaces. This BG noise reaction is unique to individual samples and varies significantly, sometimes even exceeding the true antibody-antigen reaction. As shown in Fig. 1, the BG noise reaction of samples can be easily determined in antigen non-coated wells, and compared to the OD values in antigen-coated wells (Fig. 1b). Unfortunately, this step is often skipped (Fig. 1a), and the OD values were determined in antigen-coated wells only.

The importance of determining BG noise reaction in antibody assays is shown in Fig. 2. In this experiment, serum samples from seven patients with rheumatoid arthritis (RA) were diluted to 1/500 with 2 different buffer systems: 5% BSA-0.05% Tween 20 (BSA-Tween), and ChonBlock^{TM}. In both IgG and IgA anti-liposaccharide (LPS) antibody assays, BG noise OD values obtained in antigen non-coated wells were as high as OD values in LPS-coated wells in the BSA-Tween buffer system, and consequently antigen-antibody reactions could not be distinguished from non-specific reactions in this buffer system. In contrast, BG noise OD values were significantly reduced by ChonBlock^{TM} in both IgG and IgA anti-LPS antibody assays, and the antigen-antibody reaction was clearly distinguished from non-specific false positive reaction.

Comparing the blocking ability of ChonBlock^{TM} and current blocking agents

The blocking ability of different blocking agents was compared in a high protein-binding ELISA plate (Immulon 2HD) using serum from a patient with RA and a purified human IgG solution as a control. As shown in Fig. 3a, current blocking agents are unable to prevent BG noise reaction at low serum dilutions. A buffered 100% NGS had a significant blocking effect as previously reported [1], while ChonBlock^{TM} reduced BG noise reaction even more effectively than NGS. For further quantitative comparison of the blocking ability of different blocking agents, purified human IgG was

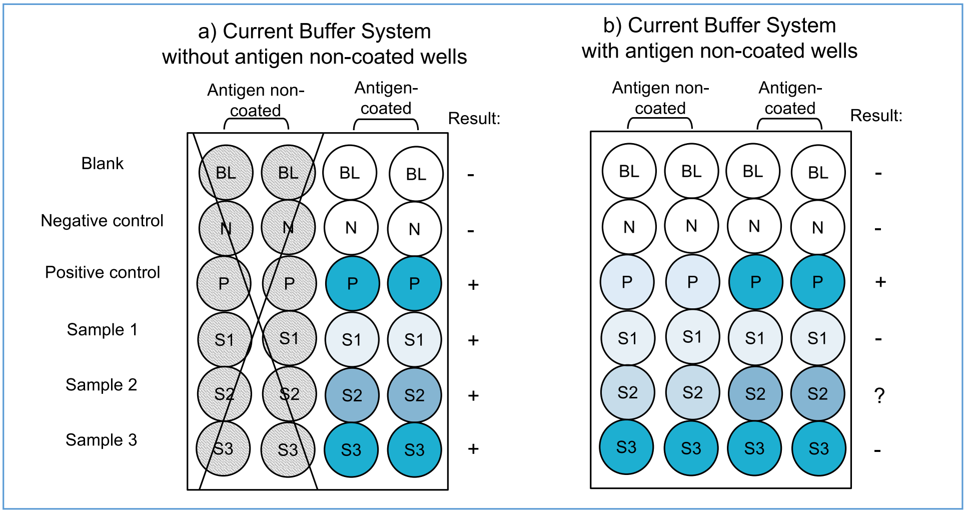

Fig. 1. Illustration of the current ELISA system for assaying antibodies. a) The BG noise reaction of individual samples can be determined in antigen non-coated wells, but this step is skipped in most cases. b) By assaying the BG noise reaction of individual samples in antigen non-coated wells and comparing to OD values in antigen-coated wells, it will be clearly revealed that current assay results are largely influenced by the BG noise reaction caused by individual test samples.

dissolved at 25 μg/ml in BSA (8–0.25%), NGS (4–0.625%), or ChonBlock™ (4–0.625%) serially diluted with TBS-0.05% Tween, and added to antigen- non-coated plain wells. As shown in Fig. 3b, the blocking ability of 0.1% ChonBlock™ is equivalent to 0.6% NGS and 5% BSA, indicating the blocking activity of ChonBlock™ is respectively six times and 40 times greater than NGS and BSA.

Comparing the BG noise reaction caused by sera from patients with RA and normal controls

Importantly, BG noise OD values are significantly higher in sera from disease groups than those of normal controls in current buffer systems such as BSA-Tween as shown in Fig. 4. Unfortunately, OD values of patient sera containing higher BG noise reaction were not corrected by subtracting the BG

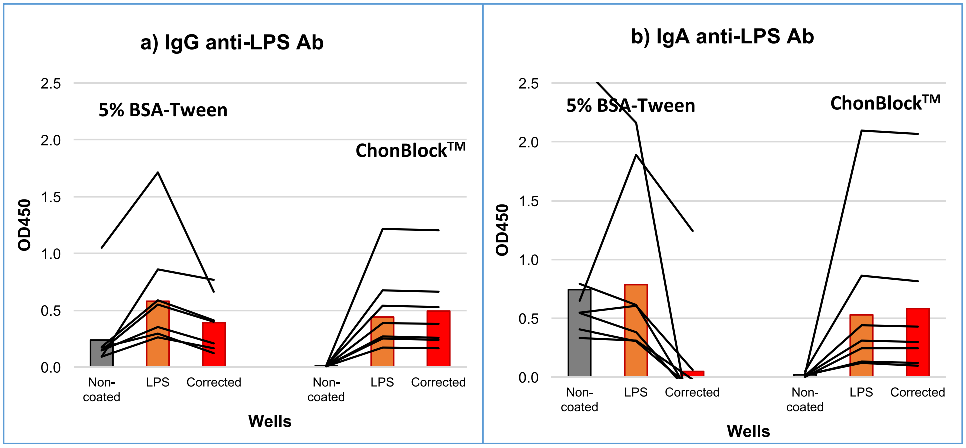

Fig. 2. Comparison of antibody assay results in different buffer systems. Serum samples from seven patients with RA were diluted at 1/500 with BSA-Tween or ChonBlock™, and assayed for (a) IgG and (b) IgA anti-LPS antibodies in antigen non-coated plain wells and LPS-coated wells.

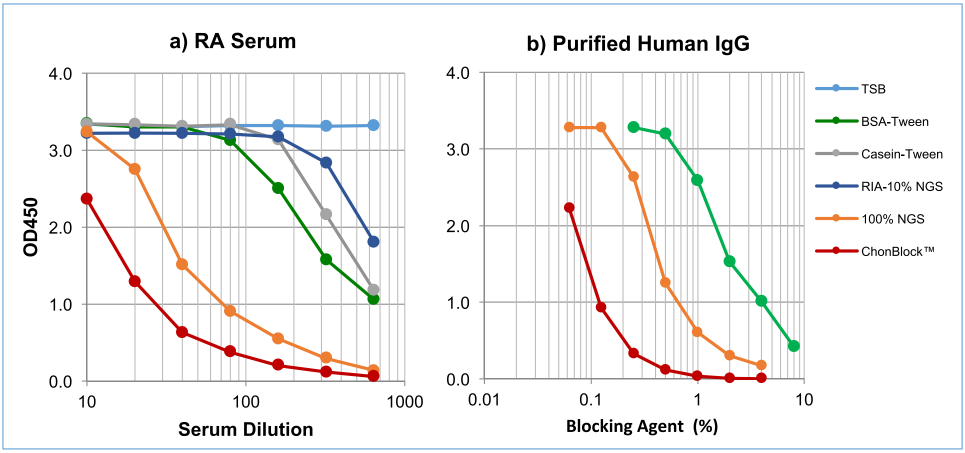

Fig. 3. Comparison of the blocking ability of different blocking agents: a) RA serum was serially diluted with various blocking buffers and added to antigen non-coated plain wells. b) Purified human IgG was dissolved at 25 μg/ml with blocking buffers, BSA (8–0.25%), NGS (0.0625–4%) and ChonBlock^{TM} (0.625–4%) serially diluted with TSB-Tween, and added to antigen non-coated plain wells. Immunoglobulin bound to the wells was determined by HRP-conjugated goat anti-human IgG antibody [3].

noise OD values, but were compared with un-corrected OD values of normal controls. Consequently, antibody levels are always deemed higher in disease groups than normal groups. To avoid this misinterpretation, it is critical to use an appropriate blocking agent to eliminate non-specific BG noise reaction, and subtract the BG noise OD values from the OD values in antigen-coated wells regardless of sample source and antigen type. Attempts to lower the OD values of normal controls, the so-called “normal range”, by decreasing the assay sensitivity using a lower concentration of secondary antibody

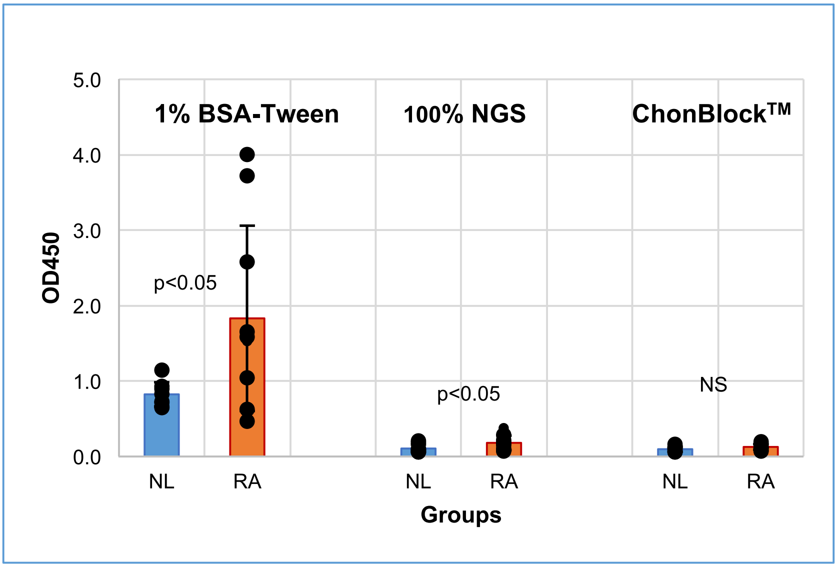

Fig. 4. Comparison of BG noise OD values of normal and RA sera in different buffer systems. Sera from 10 normal controls (NL) and 10 patients with RA were diluted to 1/200 with 1% BSA-0.05% Tween 20, Buffered 100% NGS, and ChonBlock^{TM}, and added to antigen non-coated plain wells. IgG bound to wells non-specifically was determined by goat anti-human IgG conjugated with HRP [3].

does not eliminate the BG noise reaction or improve assay accuracy, but increases the risk of obtaining false negative results. Note that the ratio of BG OD values and OD values reflecting true antibody-antigen reaction remains unchanged regardless of the assay sensitivity, as illustrated in the graphic abstract.

Other non-specific reactions involved in indirect ELISA

In addition to BG noise reaction, a variety of false positive and negative reactions are involved in indirect ELISAs as listed below. Of these, the non-specific reaction caused by secondary antibodies, which is determined in blank wells (BL), is well-recognized, and effectively prevented by current blocking agents, such as BSA, casein, non-fat milk, and others dissolved in a buffer containing Tween 20. Therefore, these blocking agents are widely used for blocking plates and diluting samples without confirming their blocking ability against different types of non-specific reactions.

False positive reactions

The principle of the ELISA system is utilizing high binding affinity of proteins to plastic surfaces. Therefore, various undesirable phenomena attributed to this principle are involved in antibody assays. It is important to recognize that the binding affinity of serum immunoglobulins to plastic surfaces is much higher than any currently used blocking agents. Immunoglobulins in heterologous serum can effectively prevent the binding of human serum immunoglobulins to plastic surfaces, as reported [1]. However, the usage of heterologous serum is very limited as discussed later. In addition, it is important to recognize that the immunoglobulin concentration in serum samples is high, in the order of mg/ml, whereas the concentration of antigen-specific antibody immunoglobulins is as low as ng or μg/ml, and the non-antibody immunoglobulins cause considerable non-specific interaction with antigen molecules. The following is a list of non-specific reactions involved in the indirect ELISA system.

a) Interactions between immunoglobulins in sample specimens and antigens: e.g. Specific interactions between immunoglobulin-fibronectin and fibronectin-collagen can occur, and as a consequence, the resulting OD value includes signal from non-antibody immunoglobulin bound to antigens [6]. NOTE: A buffered 100% NGS can block these interactions, and has been used for assaying anti-collagen antibodies [1].
b) Non-specific ionic and hydrophobic interactions between immunoglobulins and antigens are not prevented by current blocking agents such as BSA-Tween, and consequently the OD values obtained in antigen-coated wells are higher than the true OD values by 20–50% depending on the antigen [3]. NOTE: This can be determined by an inhibition test.
c) Immune recognition of blocking agents by antibodies present in sample sera: e.g. Antibodies against dietary proteins present in sample sera react with BSA, NGS, casein, and other blocking agents (Unpublished data). NOTE: An identical blocking agent must be included in both blocking and sample dilution buffers.
d) Immune recognition of contaminants in antigen preparations by antibodies present in test sera: e.g. Anti-BSA antibodies present in human sera react with minor contaminants such as BSA in insulin preparations [7].
e) Denaturation of blocking agent by ionic detergents such as sodium dodecyl sulfate (SDS). NOTE: BSA bound by SDS in RIA buffer (10 mM Tris, 1% BSA, 350 mM NaCl, 1% Triton X-100, 0.5% sodium deoxycholate, 0.1% SDS) loses antigenicity and fails to neutralize anti-BSA antibodies present in human sera. As a consequence, BSA used as blocking agent is strongly recognized by human serum anti-BSA antibodies (see Fig. 9a) [3].

False negative reactions

In addition to false positive reactions, false negative reactions are involved in the indirect ELISA system due to the inhibition of antibodies by buffer components, as listed below.

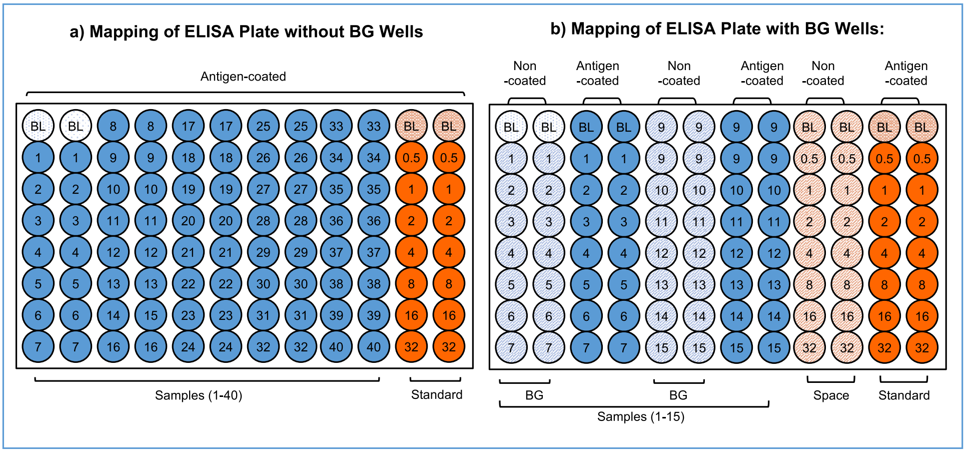

Fig. 5. Mapping of an ELISA Plate Depending on Sample Dilution. Blue: test sample added to antigen coated well, Orange: Standard added to antigen-coated wells, blue slash: test samples added to antigen non-coated wells. NOTE: If antibody positive human serum is used as a standard, it may be necessary to determine the BG noise reaction in the antigen non-coated wells shown with orange slash.

Competitive inhibition of test antibodies by relevant antibodies present in heterologous animal serum in blocking and sample dilution buffers: e.g. Inhibition of human serum anti-*Escherichia coli* (*E. coli*) antibodies by *E. coli* antibodies present in animal serum (see Fig. 7b) [3].

Inhibition of test antibodies by components in sample dilution buffer, which share similar antigenic epitopes: e.g. Inhibition of human serum Cyclic Citrullinated Peptide (CCP) antibodies by components in animal serum supplemented in the sample dilution buffer, such as RIA-10% NGS buffer (see Fig. 9b) [3].

Denaturation or blocking of antigenic epitopes of target antigens by ionic detergents, such as SDS, in a sample dilution buffer: NOTE: SDS strongly binds proteins, denatures the conformational structure, and inactivates biological activity.

NOTE: The sandwich ELISA system is widely used to measure antigens. In this assay system, no BG noise reaction observed in indirect ELISA systems is involved, because the detection antibodies used are specific to the target antigen and do not react with immunoglobulins in sample specimens. However, instead of BG noise reaction, undesirable immune recognition of capture antibodies by natural antibodies in sample specimens is often observed. For example, mouse and rat monoclonal and polyclonal antibodies used as antigen-capture antibodies will be recognized by antibodies against heterologous immunoglobulins present in human sera, which cross-react with various species of immunoglobulins. Although ChonBlock™ does not prevent this reaction, ChonBlock™ may be applied to the sandwich ELISA system to reduce other types of non-specific reactions. However, a sandwich ELISA is beyond the scope of this article, and further studies are required.

Tips for successful assay of antibodies by an ELISA

To accurately assay antibodies in human and animal sera, it is critical to 1) determine the BG noise reactions caused by individual samples in antigen-uncoated plain wells as shown in Fig. 5, 2) confirm the assay accuracy and reliability by inhibition tests in individual buffer systems, and to 3) aliquot appropriate amounts of individual reagents into multiple vials and store at 4 °C or −20 °C to be used across experiments to avoid unnecessary variation between individual assays. Based on detailed studies of the false positive and negative reactions involved in ELISA (3), the following are tips to successfully assay for antibodies with consistent reproducibility and accuracy.

Plate mapping

ELISA plates should be mapped prior to running the ELISA to determine (1) Blank (BL) values, (2) Background (BG) noise reaction values of individual samples and (3) antigen-antibody reaction values as shown in Fig. 5. In cases where the sample dilution is more than 1/1000, ChonBlock™ allows for the mapping shown in Fig. 5a. It does not require antigen non-coated wells because the BG noise values will be negligible. However, if the sample dilution is less than 1/500, it is necessary to determine the BG noise reaction of individual samples in antigen non-coated wells as shown in Fig. 5b.

Inhibition test

Unfortunately, large amounts of ELISA data are published without confirming the accuracy of the individual assay system. Consequently, assay results containing BG noise reaction values are considered true antibody-antigen reaction values, as described later. Furthermore, it is not well recognized that approximately 20–50% of the OD values obtained using BSA-Tween and BSA-Triton-X 100 buffer systems are attributed to the non-specific protein-protein interactions between immunoglobulins and antigen molecules [3]. Therefore, it is critical to confirm the accuracy and reliability of individual assay systems with an inhibition test. The following is an appropriate protocol to perform an inhibition test.

1) Dissolve the antigen at 400 μg/ml in an appropriate Blocking/Sample Dilution Buffer, then serially dilute to make 200, 100, 50, 25, 12.5, 6.3, and 0 μg/ml solutions.
2) Prepare 2× concentration of antibody solution in the same buffer.
3) Mix equal volumes of antigen and antibody solutions, and incubate at room temperature for 2 h or at 4 °C overnight.
4) Add 100 μl of the mixed solution to both antigen non-coated and antigen-coated wells, and run the assay to determine the antigen-specific inhibition.
5) Calculate the corrected OD values by subtracting the BG OD values in antigen non-coated wells from the OD values in antigen-coated wells. Then, calculate the inhibition percentage by comparing the OD values in wells containing inhibition antigen with OD values in the control wells without inhibition antigen (see Figs. 8 and 10).

Reagents

Buffer systems

To accurately assay antibodies, it is important to eliminate false positive BG noise reactions caused by the sample itself. Because no current blocking agents, except buffered 100% heterologous serum, can block this BG noise reaction, we investigated all types of non-specific reactions involved in ELISA and reported that a new blocking buffer, "ChonBlock™", eliminates this BG noise reaction and other false positive and negative reactions involved in ELISA [3]. The blocking effect of ChonBlock™ is 40 times greater than BSA, as shown in Fig. 3. This buffer system can be used to assay antibodies against virtually all types of antigens including potential pathogenic environmental agents and auto-antigens for human and animal sera [3]. NOTE: ChonBlock™ cannot be used for assaying anti-collagen antibodies as described above.

Antigen

If the antigen is not stable in solution, dissolve the antigen at 5–10 mg/ml in 5% sucrose, aliquot 0.1 ml into vials, lyophilize, and store at −20 °C. Dilute 1:100 with a buffer for plate coating. In general, lyophilized antigens are stable at −20 °C.

Standard

Choose appropriate antibodies, such as a monoclonal antibody, polyclonal antibodies or antibody-positive serum, and then predetermine antibody titer. Dilute the antibody (for example: 320 units/ml)

with 4% BSA dissolved in 0.02 M Tris-0.15 M NaCl buffer, pH 7.2, containing 5% sucrose. Aliquot 0.1 ml of the standard solution into vials, lyophilize, and store at −20 °C. Reconstitute the stock standard with 1 ml of a blocking/sample dilution buffer (32 units/ml).

Secondary antibody conjugated with enzyme

Prepare secondary antibody in 50% ethylene glycol containing 1% BSA. Optimize secondary antibody concentration to obtain sufficient assay sensitivity, while keeping the blank OD value low. If the final dilution used for assay is 1/1000 or 1/2000, make a 1/5 or 1/10 solution from the stock solution with the ethylene glycol-EG-BSA solution, then aliquot 50 μl into vials. Store at −20 °C. Dissolve one vial of the secondary antibody in 10 ml secondary antibody dilution buffer before use. NOTE: Do not use glycerol because contaminants in glycerol may gradually inactivate the peroxidase conjugated to secondary antibodies during storage.

Washing buffer

Prepare a 20× PBS-1% Tween 20 stock solution and store at room temperature. Prepare fresh 1× wash buffer by diluting with endotoxin-free distilled water just before use. NOTE: Bacteria will not grow in a 20× buffer with high salt concentration.

Calculation of antibody titers

1) Average the duplicate OD values for the blank (BL), standards, and test samples.
2) Subtract the averaged BL values from the averaged OD values of all wells of test samples and standards.
3) If the BG noise reaction of individual samples was determined, subtract the corrected BG value in non-coated wells from the corrected OD value in antigen-coated wells of individual test samples.
4) Plot the OD values of standards against the units/ml. A log/log plot will linearize the data as shown in Fig. 6.
5) The concentration of antibodies (units/ml) in test samples can be calculated using regression analysis.

Comparison of assay results using ChonBlock™ and other buffers

Antibody assay results vary significantly depending on the buffer system used, because different buffer components contribute to different non-specific reactions. To demonstrate the importance of eliminating all types of non-specific reactions, human serum IgG anti-*E. coli* and anti-CCP antibodies were assayed in different buffer systems under the same conditions.

*Anti-*E. coli *antibody assay results in different buffer systems: preventing non-specific immunoglobulin-antigen interactions*

Non-specific interactions between antigenic molecules and serum immunoglobulins are not well-recognized. This non-specific reaction varies significantly depending on the type of antigen and serum dilution, and will account for 20–50% of the OD value in assay data [3]. Unfortunately, the higher OD values caused by this non-specific reaction are misunderstood to mean that the assay sensitivity is higher in these buffer systems. To illustrate this phenomenon, human serum antibodies against *E. coli* particles were assayed in 3 different buffer systems. As shown in Fig. 7, divergent dilution curves were observed depending on the buffer systems used. In the 1% BSA-Tween (Fig. 7a), the OD values in *E. coli*-coated wells were significantly higher than those seen in other buffer systems (Fig. 7b and c). On the other hand, 100% NGS strongly inhibited the binding of antibodies to *E. coli* (Fig. 7b), indicating that anti-*E. coli* antibodies were competitively inhibited by similar antibodies in 100% NGS. In contrast, ChonBlock™ provided a clear dilution curve with low BG as shown in Fig. 7c.

To understand the discrepancies among assay results in different buffer systems (as seen in Fig. 7), an inhibition test for human serum anti-*E. coli* antibodies were performed in 1% BSA-Tween and

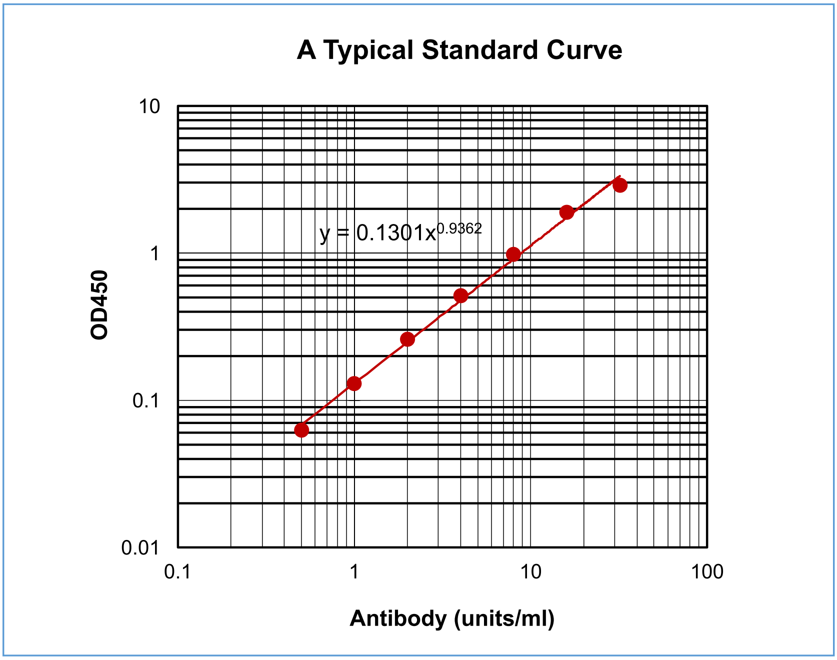

Fig. 6. A typical standard curve in the ChonBlock™ buffer system: Normal human serum was serially diluted from 32 to 0.5 units/ml with ChonBlock™ Blocking/Sample Dilution Buffer, and added to *E. coli* LPS-coated wells. Antibody bound to LPS was determined by HRP-conjugated anti-Human IgG antibody diluted to 1/10,000 with ChonBlock™ Secondary Antibody Dilution Buffer.

ChonBlock™ in *E. coli*-coated wells and antigen non-coated wells (Fig. 8). In the 1% BSA-Tween buffer, inhibition of the antibodies by *E. coli* was approximately 70% (Fig. 8a), whereas more than 90% was inhibited in the ChonBlock™ buffer system (Fig. 8b). This demonstrated that BSA does not effectively prevent the non-specific interaction of *E. coli* particles with serum immunoglobulins.

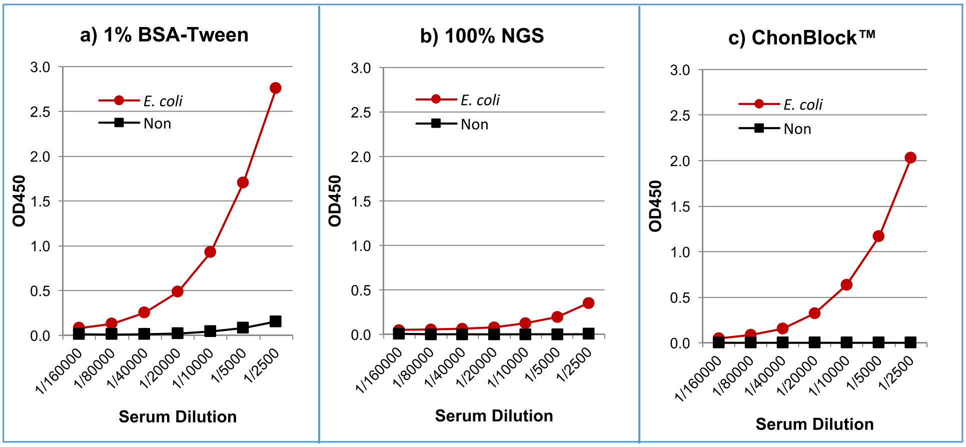

Fig. 7. Comparison of anti-*E. coli* antibody assay results in different buffer systems. Normal human serum was diluted serially with a) 1% BSA-Tween, b) 100% NGS and c) ChonBlock™, and added to antigen non-coated wells and *E. coli*-coated wells. Antibodies bound to *E. coli* were assayed by goat anti-human IgG (Fc-specific) antibody conjugated with HRP [3]. NOTE: BG noise OD values even in 1% BSA-Tween buffer are relatively low in this assay, because of the high serum dilution.

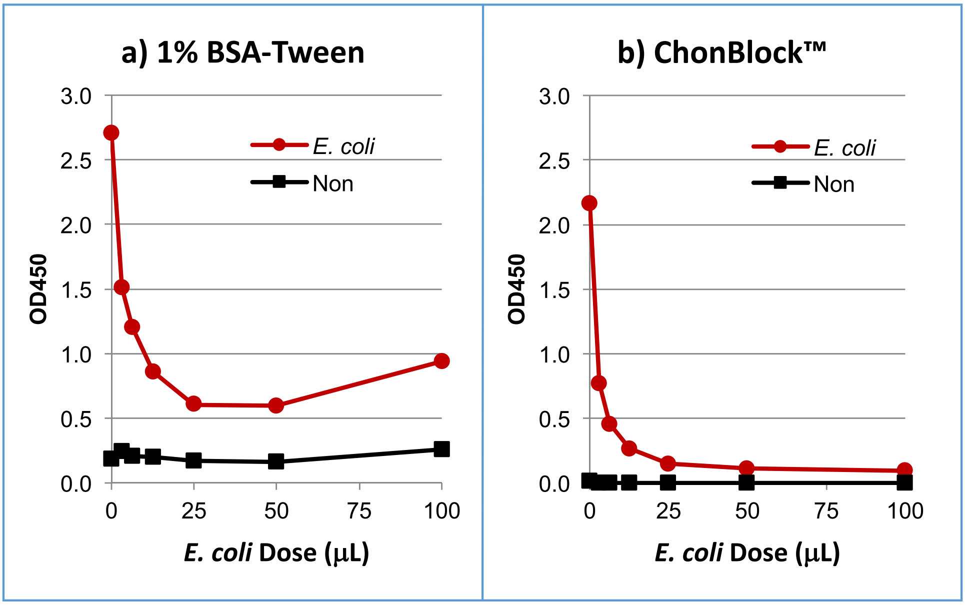

Fig. 8. Inhibition test of anti-*E. coli* antibodies in the BSA-Tween and ChonBlock™ buffer systems. An *E. coli* suspension was aliquoted into micro-centrifuge tubes and mixed with a normal human serum diluted at 1/100 with 1% BSA-Tween or ChonBlock™. After incubation at 4 °C overnight, sample solutions were further diluted to 1/2500 with the respective buffers and added to antigen non-coated and *E. coli*-coated wells [3].

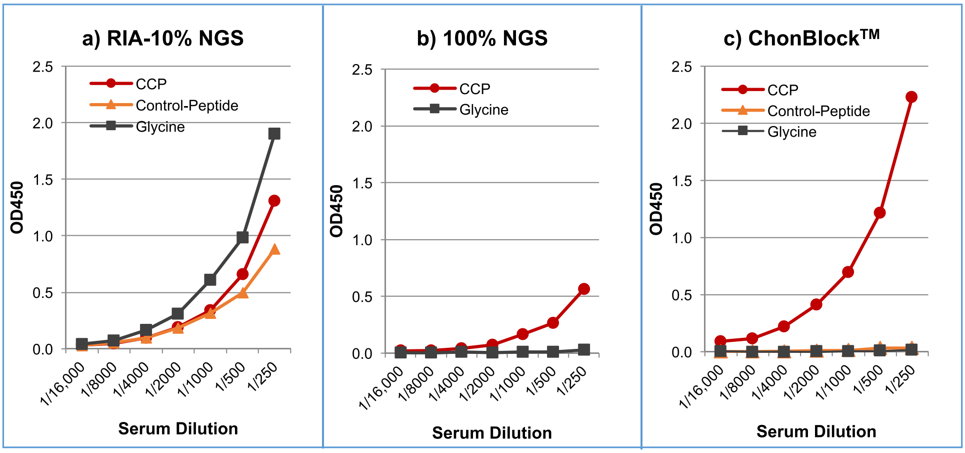

Fig. 9. Comparison of anti-CCP antibody assay results in three different buffer systems. Serum from a patient with RA was serially diluted with buffers, a) RIA-10% NGS, b) 100% NGS and c) ChonBlock™, and added to glycine (Gly)-, control peptide- and CCP-coupled wells. All wells were pre-blocked with 2% BSA, 100% NGS, and ChonBlock™ respectively [3]. NOTE: In the RIA-10% NGS, BSA used for blocking was strongly recognized by anti-BSA antibodies in sample serum because BSA-bound with SDS in this buffer lost the antigenicity to neutralize the anti-BSA antibodies in human serum specimens. In addition, NGS in RIA-10% NGS buffer partially inhibits anti-CCP antibodies.

Anti-CCP antibody assay results in different buffer systems: preventing high BG noise reaction and non-specific immunoglobulin-antigen interactions

Cyclic citrullinated peptide (CCP) antibodies in serum from a patient with RA were assayed in three different buffer systems under the same conditions in a Costar covalent plate (Fig. 9). In this experiment, glycine-coupled wells were used for determining the BG noise OD values instead of antigen non-coated wells. In RIA-10% NGS, identical dilution curves were observed in glycine-, control peptide- and CCP-coupled wells (Fig. 9a), indicating that RIA-10% NGS failed to block any type of non-specific reaction. On the other hand, CCP antibodies were significantly inhibited in 100% NGS (Fig. 9b), indicating that NGS contains components which share identical or similar antigenic epitopes with CCP. In contrast, a clear dilution curve with low BG noise reaction was achieved using the ChonBlock™ system (Fig. 9c).

To determine the specificity and accuracy of anti-CCP antibody assay results in these buffer systems, inhibition tests were performed in RIA-10% NGS, Triton X, and ChonBlock™ buffer systems. The inhibition test data for anti-CCP antibodies in RIA-10% NGS was inconclusive due to the high BG noise reaction and the inhibition of anti-CCP antibodies by NGS (Fig. 10a). On the other hand, Triton X buffer provided in a commercially available anti-CCP assay kit prevented BG noise reaction effectively, but only 53% CCP inhibition was achieved (Fig. 10b). This indicates that approximately 50% of the assay data reflects non-specific serum immunoglobulin-CCP interaction in this buffer system. In contrast, 92% inhibition was obtained in the ChonBlock™ buffer system (Fig. 10c). These results indicate that false positive reactions caused by non-specific interactions between antigens and serum immunoglobulins were underestimated in the past.

Comparison of anti-CCP antibody assay results in the RIA-10% NGS and ChonBlock™ buffer systems

Based on the inhibition test results, ChonBlock™ effectively prevents various types of non-specific reactions in antibody assays. To confirm the advantages of using the ChonBlock™ buffer system, anti-CCP antibodies were assayed in RIA-10% NGS, which was widely used for assaying anti-CCP antibodies, and compared with assay results from ChonBlock™ buffer system (Fig. 11). In the RIA-10% NGS buffer,

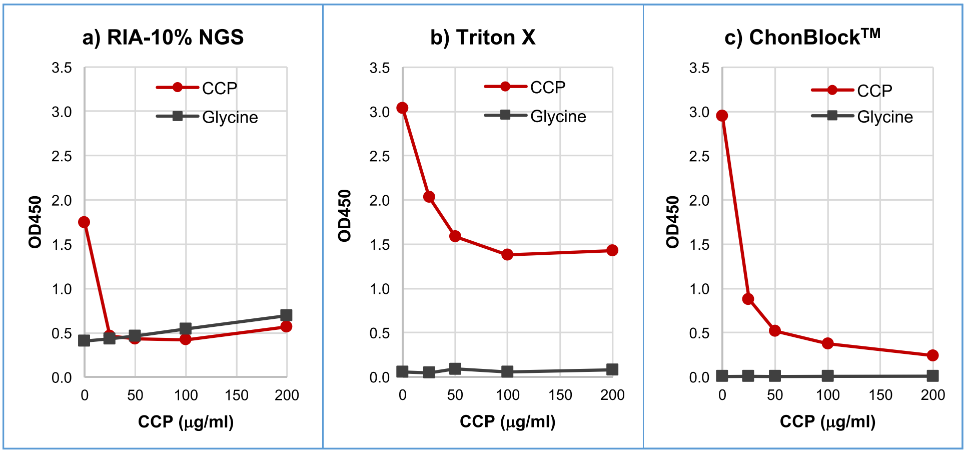

Fig. 10. Inhibition test of anti-CCP antibodies in 3 different buffer systems. RA serum was diluted at 1/800 in a) RIA-10% NGS, b) BSA-Triton X and c) ChonBlock™, respectively, and 0.8 ml of this solution was mixed with 0.2 ml of CCP solution (0.1–1 mg/ml in distilled water). After incubation at 4 °C overnight, the sample solutions were added to glycine- and CCP-coupled wells, and incubated at room temperature for 2 h. HRP-conjugated goat anti-human IgG antibodies diluted to 1/10,000 in 2% NGS was added to all wells and reacted at room temperature for 2 h. NOTE: Glycine-coated wells were used for determining BG noise reaction [3].

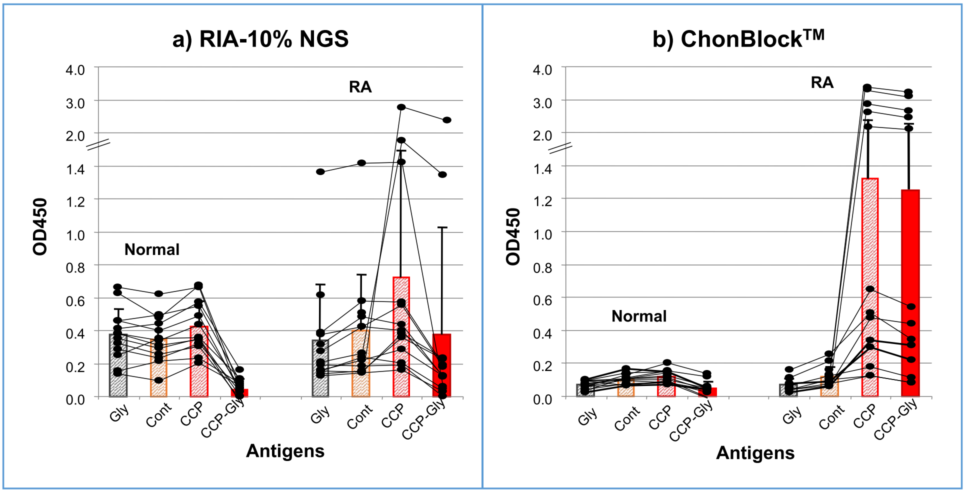

Fig. 11. Comparison of anti-CCP antibody assay results using a) RIA 10% NGS and b) ChonBlock™ buffer systems. Sera from 13 normal controls and 13 patients with RA were diluted 1/500 with the respective buffers and added to glycine-, control peptide-, and CCP-coupled wells [3].

similar OD values were observed in glycine- (Gly), control peptide- (Cont), and CCP-coupled wells for both normal and RA sera. If the OD values obtained in CCP-coupled wells are corrected by subtracting the BG noise OD values obtained in Gly-coupled wells (CCP-Gly), no significant difference was observed between normal control and RA groups, and only two RA samples were considered antibody positive (Fig. 11a). Using ChonBlock™, BG noise OD values in Gly-coupled and control peptide-coupled wells were equally low in both normal and RA sera, and distinguishable from the robust OD values in CCP-coupled wells (Fig. 11b). The results demonstrated that two out of 13 normal controls and 11 out of 13 RA patients were anti-CCP antibody positive.

Summary

It is critical to reduce or eliminate non-specific reactions in any immunological assay system to avoid false positive and negative results. It is especially important to consider BG noise reaction caused by the hydrophobic binding of immunoglobulins in sample specimens to solid surfaces in indirect ELISA systems for assaying serum antibodies, particularly at low sample serum dilutions. In addition, non-specific binding of immunoglobulins to antigenic molecules by protein-protein interactions also creates unpredictable degrees of false positive reactions. We encourage all ELISA users to take into consideration the various types of non-specific reactions involved in the indirect ELISA system, and use appropriate buffer systems and proper ELISA design to prevent further misuse of the ELISA technique and misinterpretation of serological antibody assay data.

Authors' contribution

KT designed the experiments, coordinated their completion, acquired, analyzed and interpreted the data, and drafted this manuscript. JC, BM, and TW confirmed the data by conducting related, but independent experiments, and contributed in the preparation of this manuscript. TW conducted the recovery tests in antibody assays by ELISA and assisted in the interpretation of the data. All authors have read and approved of the final manuscript.

Human serum samples disclosure

An Institutional Review Board (IRB) exemption was granted from the Western Institutional Review Board (WIRB), Olympia, WA, USA. According to the WIRB Regulatory Affairs Department, this research project met the conditions for exemption under 45 CFR 46.101(b)(4). All experiments were conducted with informed consent from patients.

References

[1] K. Fujii, M. Tsuji, K. Murota, K. Terato, Y. Shimozuru, et al., An improved enzyme-linked immunosorbent assay of anti-collagen antibodies in human serum, J. Immunol. Methods 124 (1) (1989) 63–70.
[2] D. de Vries, A. Dekker-Saeys, E. Gyodi, U. Bohm, P. Ivanyi, Absence of autoantibodies to peptides shared by HLA-B27.5 and Klebsiella pneumoniae nitrogenase in serum samples from HLA-B27 positive patients with ankylosing spondylitis and Reiter's syndrome, Ann. Rheum. Dis. 51 (6) (1992) 783–789.
[3] K. Terato, C. Do, D. Cutler, T. Waritani, H. Shionoya, Preventing intense false positive and negative reactions attributed to the principle of ELISA to re-investigate antibody studies in autoimmune diseases, J. Immunol. Methods 407 (2014) 15–25.
[4] K. Terato, C. Do, H. Shionoya, Slipping through the cracks: linking reduced immune function and intestinal bacteria imbalance to the etiology of rheumatoid arthritis, Autoimmune Dis. 2015 (2015).
[5] K. Terato, C. Do, J. Chang, T. Waritani, Commentary: preventing further misuse of the ELISA technique and misinterpretation of serological antibody assay data, Vaccine 34 (39) (2017) 4643–4644, doi:http://dx.doi.org/10.1016/j.vaccine.2016.08.007 Epub 2016 Aug 6.
[6] M. Mannik, S. Kapil, C.E. Merrill, In patients with rheumatoid arthritis IgG binding to denatured collagen type II is in part mediated by IgG-fibronectin complexes, J. Immunol. 158 (3) (1997) 1446–1452.
[7] A. Williams, R. Curnock, C. Reed, P. Easton, S. Rokni, et al., Anti-BSA antibodies are a major cause of non-specific binding in insulin autoantibody radiobinding assays, J. Immunol. Methods 362 (1–2) (2010) 199–203.
