# Supplementary figures and images for: Contribution of bacterial pathogens to evoking serological disease markers and aggravating disease activity in rheumatoid arthritis
Source: PLoS One. 2018 Feb 6;13(2):e0190588. doi: 10.1371/journal.pone.0190588 (PMC5800560; doi:10.1371/journal.pone.0190588)

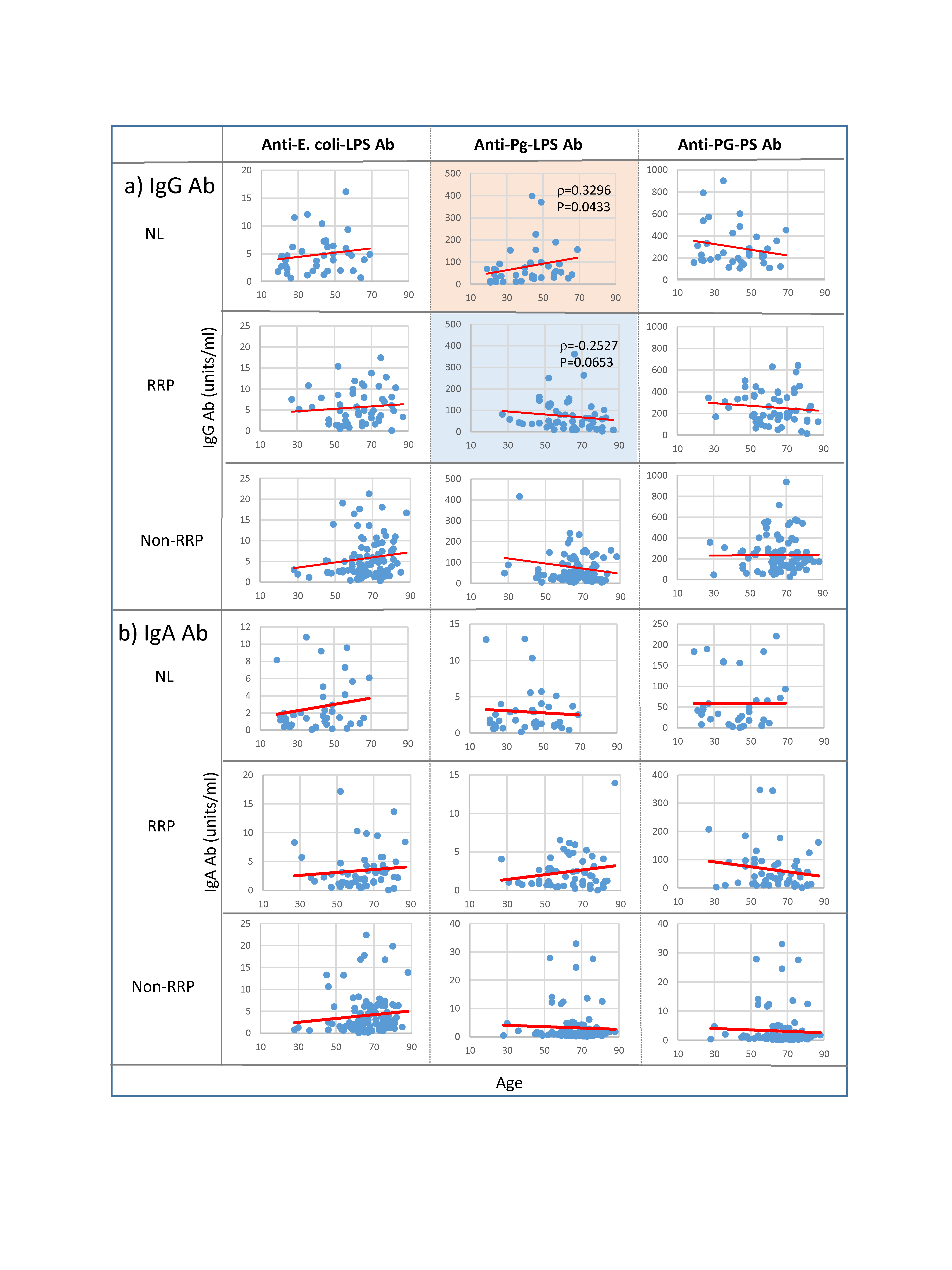

Supplement: S1 Fig — IgG and IgA antibody levels against E. coli-LPS, Pg-LPS and PG-PS were determined in sera from 38 NL controls, 54 patients with RRP and 101 patients with non-RRP, and plotted against age. Antibody levels against these pathogens were not affected by age in both NL and RA groups, except IgG anti-Pg-LPS antibody, which increased with age in the NL controls, whereas tended to decrease in the RRP group. NOTE: Pink: significant correlation at p<0.05, Blue: trending toward correlation at 0.05≦p<0.15, No color: no correlation. (TIF) [file pone.0190588.s002.tif]

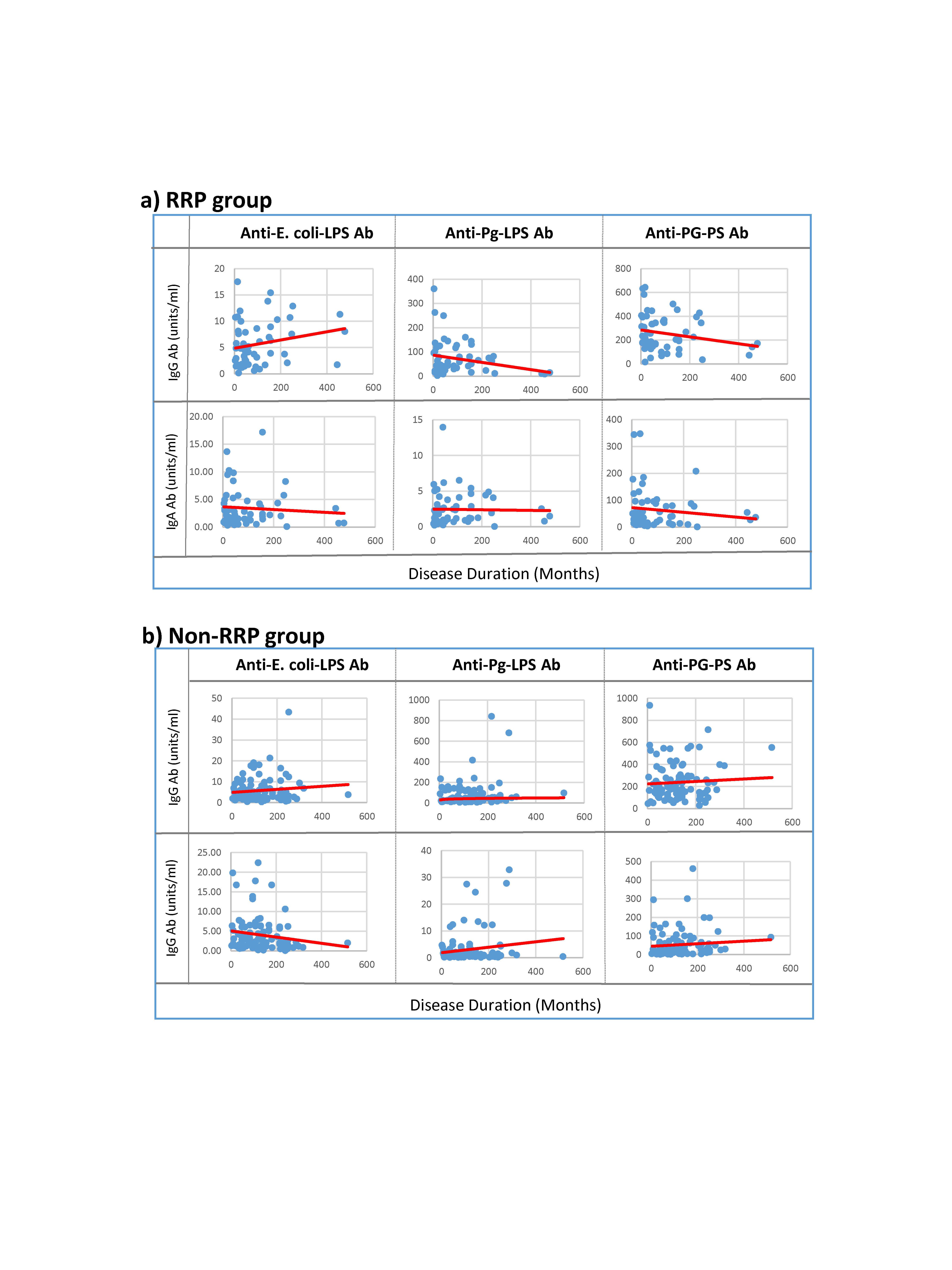

Supplement: S2 Fig — IgG and IgA antibody levels against E. coli-LPS, Pg-LPS and PG-PS were determined in sera from 54 patients with RRP and 101 patients with non-RRP, and plotted against disease duration (months). No apparent antibody level change associated with disease duration was observed. This evidence was confirmed in a separate study on the effect of therapeutics on IgG and IgA antibody responses in patients with RA. (TIF) [file pone.0190588.s003.tif]

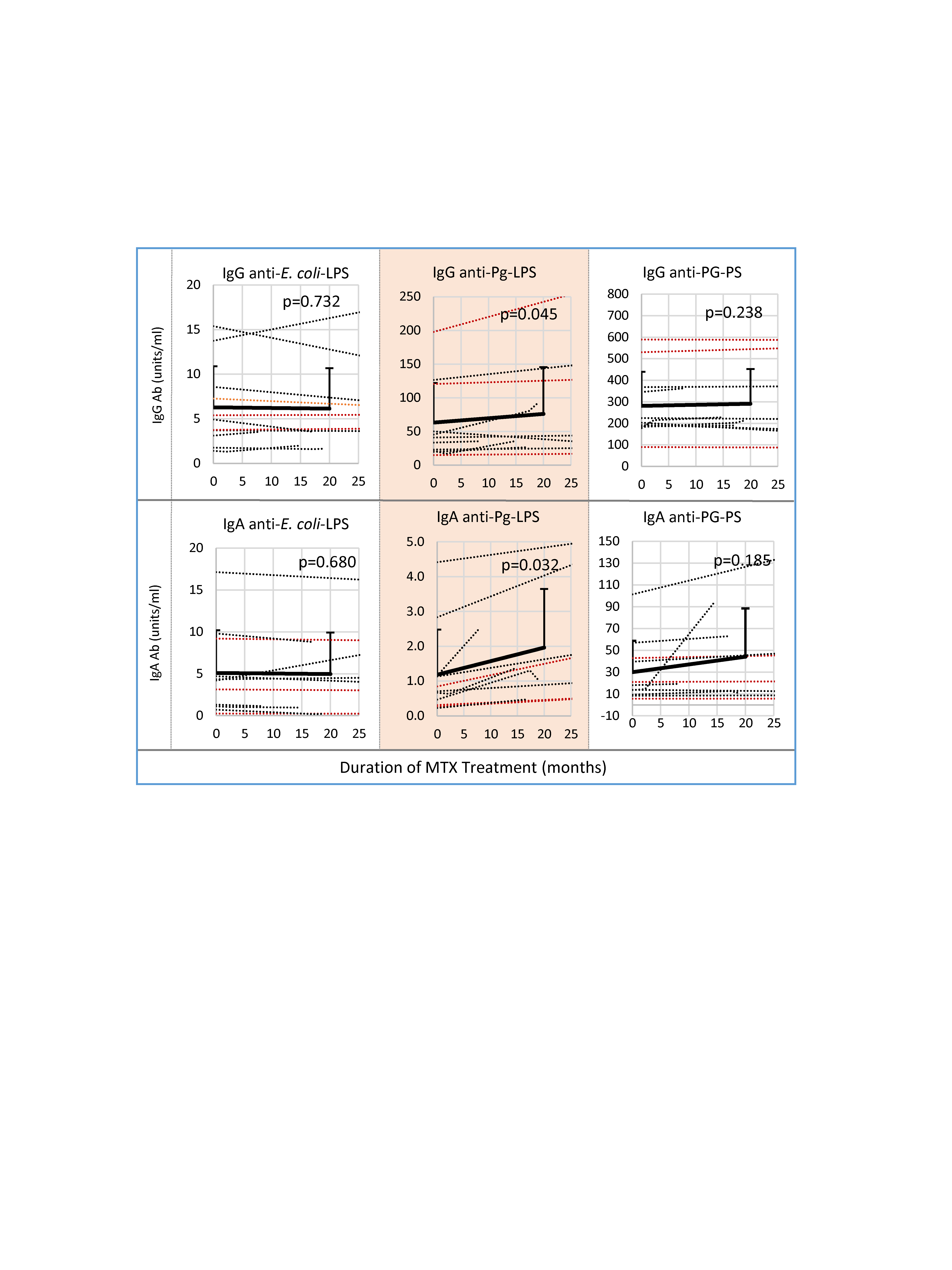

Supplement: S3 Fig — IgG and IgA antibody levels against E. coli-LPS, Pg-LPS and PG-PS were determined in sera collected multiple times from 7 patients with RRP and 3 patients with non-RRP, who were treated with MTX for 13 to 113 months. MTX treatment did not affect anti-E. coli-LPS and PG-PS, but apparently increased IgG and IgA antibody responses to Pg-LPS. NOTE: dotted black line: RRP, dotted red line: non-RRP, solid black line: an average ± SD at 20 months. Pink: significant correlation at p<0.05. No color: no correlation. (TIF) [file pone.0190588.s004.tif]

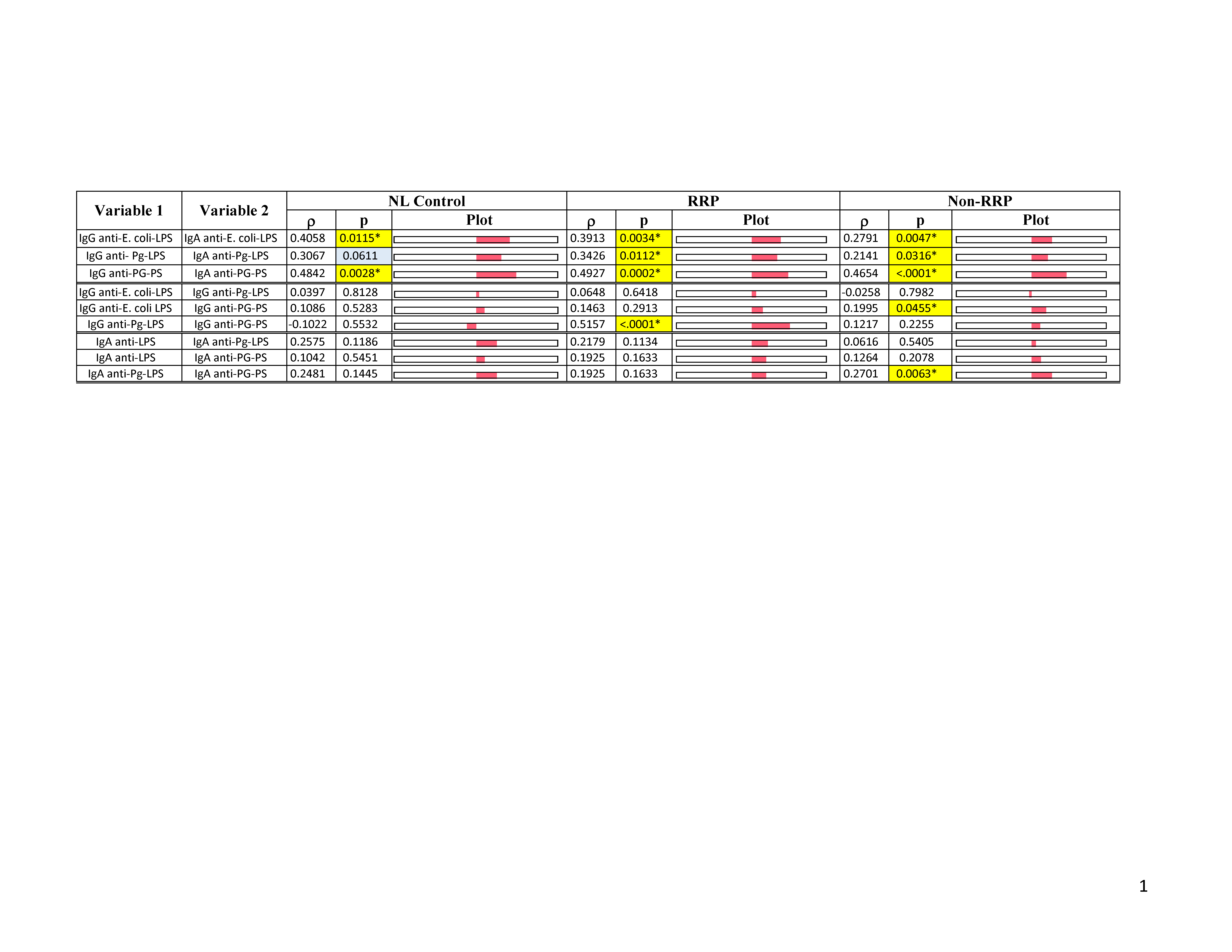

Supplement: S4 Fig — IgG and IgA antibody levels against E. coli-LPS, Pg-LPS and PG-PS were determined in sera from 38 NL controls, 54 patients with RRP and 101 patients with non-RRP, and analyzed potential correlation between antibody responses against individual pathogens by Spearman non-parametric rank correlation analysis. IgG antibody levels against individual pathogens correlated or tended to correlate with IgA antibody levels in both NL and RA. By contrast, IgG antibody levels to individual pathogens did not correlate with IgG antibody levels against other pathogens in NL controls, indicating that IgG antibody responses to individual pathogens are unrelated independent event. However, IgG anti-Pg-LPS antibody levels correlated with IgG anti-PG-PS antibody levels in RRP, and IgG anti-E. coli-LPS antibody levels correlated with IgG anti-PG-PS antibody levels in non-RRP, indicating antibody responses to Pg-LPS and PG-PS in RRP and antibody responses to E. coli-LPS and PG-PS in non-RRP are orchestrated. NOTE: Plot: Visual display for positive and negative “ρ” value of Spearmen correlation coefficient. Cells highlighted with yellow indicate significant correlation at p<0.05, and blue indicate a trend at p<0.0.1. (TIF) [file pone.0190588.s005.tif]
