# Supplementary material for: Contribution of bacterial pathogens to evoking serological disease markers and aggravating disease activity in rheumatoid arthritis
Source: PLoS One. 2018 Feb 6;13(2):e0190588. doi: 10.1371/journal.pone.0190588 (PMC5800560; doi:10.1371/journal.pone.0190588)
Supplement: S1 File — (PDF) [file pone.0190588.s006.pdf]

試験計画書

試験題名：抗リウマチ薬の消化管免疫に及ぼす影響

Effects of Disease-Modifying and Biological Drugs on Intestinal Immunity in Patients with RA

UMIN-CTR 臨床試験登録番号：

試験実施者・所属機関名と氏名：

試験調整医師・責任医師：片山整形外科リウマチ科クリニック　片山　耕

血清検体測定責任者:Chondrex Inc. 寺戸 国昭、割谷 孝貴

血清検体測定責任者:アサマ化成株式会社 塩野谷 博

１．目的

消化管は栄養の吸収器官であると同時に、 消化管病原微生物やそれらの産生する毒素の侵入から生体を防御する物理的なバリヤー機能とともに生体防御のためのもっとも重要な免疫臓器である。

関節リウマチ（RA）の薬物治療は主に免疫と炎症を抑えることを目的として使用されているが、その結果、特に免疫抑制作用の強い生物学的製剤は感染抵抗性を低めることが危惧されている（医薬品添付文書参照)。一方、生体防御機能の面から消化管免疫能の重要性が指摘されているが、RA の薬物療法が消化管免疫に与える影響についての系統的な研究は未だ乏しい。今後の RA のより良い治療法を確立するため、RA 治療薬物の消化管免疫に及ぼす作用について検討する必要性が考えられる。そこで、抗リウマチ薬（DMARDs）や生物学的製剤（BIO）による治療を受けている RA 患者において、通常の RA 病態を把握する検査に加え、消化管免疫機能の指標として腸内細菌菌体成分に対する血清抗体、消化管バリヤー機能、炎症性サイトカイン、酸化ストレスを指標とした試験を行い、ＲＡ患者の消化管免疫の状態を知るとともに治療法の改善に資する。

２．試験項目と実施機関

消化管免疫のパラメーターとして以下の測定を行う。

１．消化管免疫機能の指標として抗ペプチドグリカン抗体、抗大腸菌抗体、抗ﾘﾎﾟﾎﾟﾘｻｯｶﾗｲﾄﾞ(LPS)抗体（コンドレックスインク）

２．消化管バリヤーの指標として LPS 血中濃度（アサマ化成㈱）と LPS 糞便濃度

３．患者の炎症状態の指標として CRP（片山整形外科リウマチ科クリニック)，炎症性サイトカインとして TNF，IL-6（コンドレックスインク）

４．RA に特徴的な指標として抗 CCP 抗体（コンドレックスインク）
５．酸化ストレス（アサマ化成㈱）

３．対象とするＲＡ患者と対照健常人

患者は ACR2011 年新基準により RA と診断された患者である。

以下に示した３群のＲＡ患者（各２０例以上）から治療前後（または治療中）に 6 か月以上の間隔で２回以上採血し、その血清を分離し、測定に供する。

ＲＡ患者の治療に使用する薬剤と用量を以下に示す。

抗リウマチ剤

1. ブシラミン　経口（50－200mg/日）
2. サルファサラジン　経口（250－1000mg/日）
3. タクロリムス　経口（0.5－3mg/日）
4. メソトレキサート　経口（2－16 mg/週）
5. ミゾリビン　経口（25-150mg/日）

生物学的製剤

1. インフリキシマブ　点滴(3mg－10mg/kg/ 8 週)
2. アダリムマブ　皮下注（40mg/2 週）
3. トシリズマブ　点滴（8mg/kg/4 週)、皮下注（162mg/2 週）
4. エタネルセプト　皮下注（25-50mg/週）
5. ゴリムマブ　皮下注（50mg または 100mg/4 週）
6. アバタセプト　皮下注（125mg/週）
7. セルトリズマブ　皮下注（400mg/4 週）

対照として、ＲＡ患者群と年齢のマッチした 20 例の健常人より６カ月以上の間隔で採取した血清を用いる。健常人は片山整形外科リウマチ科クリニックおよびアサマ化成㈱関係者よりリクルートする。

１．未治療の患者の DMARDs 治療前後
２．DMARDs 治療中の患者
３．DMARDs 抵抗性の患者の Bio 治療前後
４．健常人（対照）

４．血清の採取と送付

通院の際の採血の5㎖を本試験の検体として、血清を分離する。血清は患者番号を付して凍結し、アサマ化成㈱に送付する。

送付先：

〒103－0001 東京都中央区日本橋小伝馬町20-3

電話03-3661-6282 ファックス03-3661-6285
アサマ化成株式会社 北村香織 kitamura@asama-chemical.co.jp

5．研究に参加することにより期待される利益及び不利益

個々の被験者の消化管免疫の状態についての情報が得られる。その状態に問題があれば、問題解決の方策を可能な限り治療に反映させる。

6．個人情報保護の方法

消化管免疫の測定用試料となる血清はカルテ番号と採血日に対応する番号を付し、検体識別番号とすることにより、連結可能匿名化する。

7．インフォームド・コンセントのための手続き

説明文書（別添）により説明し、文書で同意を得る。

8．臨床研究に係る資金源、起こり得る利害の衝突及び研究者等の関連組織との関わり（利益相反）

臨床試験に必要とする経費は原則として、各研究者の所属機関が負担する。

9．被験者の費用負担

被験者の費用負担、謝金はない。

10. 試験の倫理委員会審査

旭川医科大学倫理委員会に臨床審議申込書をもって申請する。

11. 臨床試験登録

大学病院医療情報ネットワーク（UMIN）に申請した。登録番号はUMIN000012200である。

12. 成果の帰属と公表

この研究の成果は全ての試験実施者と所属機関に帰属にし、日本リウマチ学会での発表並びに論文審査のある学術専門誌に投稿することとする。

13．試験期間

試験開始（倫理委員会認定日）より2年間後を試験終了日とする。
