# Supplementary material for: Contribution of bacterial pathogens to evoking serological disease markers and aggravating disease activity in rheumatoid arthritis
Source: PLoS One. 2018 Feb 6;13(2):e0190588. doi: 10.1371/journal.pone.0190588 (PMC5800560; doi:10.1371/journal.pone.0190588)
Supplement: S2 File — (DOCX) [file pone.0190588.s007.docx]

**Clinical Study Plan**

**Title: Effects of Disease-Modifying and Biological Drugs on Intestinal Immunity in Patients with RA**

Trial Registration Number: UMIN000012200

**Investigators and Affiliations**

Principal Investigator: Kou Katayama, Katayama Orthopedic Rheumatology Clinic, Asahikawa, Hokkaido, Japan

Co-investigators:

Serological Analysis 1: Kuniaki Terato & Takaki Waritani, Chondrex Inc., Redmond, WA. USA

Serological Analysis 2: Hiroshi Shionoya, Asama Chemicals. Co. Ltd., Tokyo, Japan

**Objective**

The gastrointestinal immune system is the most crucial defense system to protect the host from large amounts of foreign substances including bacteria and their toxins by physiological and immunological barrier function. However, immune-suppressive therapeutics are widely used for treatment of patients with rheumatoid arthritis to reduce inflammatory reaction. Therefore, we consider that it is indispensable to study possible adverse effects of these therapeutics on intestinal immune function, because it is highly likely that a variety of disease modifying drugs (DMARDs) and Biological therapeutics may suppress the host’s immune function and increase susceptibility to infectious agents. Unfortunately, the adverse effects of these therapeutics have not been extensively studied yet. To develop better treatment strategies and methods, we plan to study the effects of therapeutics on patient immune function by determining serum antibody responses to potential environmental pathogens, mucosal barrier function, serum cytokines, and oxidative stress in patients with RA before and after treatment with DMARDs and biological therapeutics.

**List of clinical test markers and implementing organization**

2-1 Markers for intestinal immune function: Serum antibody levels against peptidoglycan-polysaccharide, E. coli, Lipopolysaccharides (Chondrex Inc.,)

2-2 Marker for mucosal barrier function: Serum LPS levels (Asama Chemicals Co. Ltd.,)

2-3 Inflammatory markers: CRP (Katayama Rheumatology Clinic) and serum TNF and IL-6 levels (Chondrex Inc.,)

2-4 RA specific marker: anti-CCP antibody (Chondrex Inc.,)

2-5 Oxidative Stress (Asama Chemicals Co. Ltd.,)

**Patients and controls**

Patients with RA diagnosed by ACR 2011 criteria will be classified into 3 groups (more than 20 patients per group) as shown below. An initial serum sample will be taken upon enrollment in the study, and every six months thereafter. A minimum of two serum samples will be collected (before and after treatment), with more samples to be collected from patients undergoing treatment for longer periods. As a control, serum from 20 normal controls will be collected twice at 6 month intervals.

Group 1: Non-treated patients before treatment with DMARDs

Group 2: Patients currently under treatment with DRARDs

Group 3: DMARDs-resistant patients before and after treatment with Biologicals

The therapeutics used for treatment of patients are listed bellows:

Anti-Rheumatic:

1. Bucillamine: Oral (50-200mg/day)

2. Sulfasalazine: Oral (250-1000 mg/day)

3. Tacrolimus: Oral (0.5- 3 mg/day)

4. Methotrexate: Oral (2-16 mg/day)

5. Mizoribine: Oral (25-150 mg/day)

Biologics:

1. Infliximab: Infusion (3-10 mg/Kg/8 weeks)

1. Adalimuma: SC (40 mg/2 weeks)
2. Tocilizumab: Infusion (8 mg/kg/4 weeks) or SC (162 mg/2 weeks)

4. Etanercept: SC (25-50 mg/week)

5. Golimumab: SC (50 or 100 mg/4 weeks)

6. Abatacept: SC (125 mg/week)

7. Certolizumab: SC (400 mg/4 weeks)

**Serum collection and transfer**

Serum samples collected at Katayama Rheumatology Clinic will be labeled with serial number and shipped with dry ice to Asama Chemicals, Tokyo, and then shipped to Chondrex Inc., USA.

**Advantages and disadvantages for patients participating in this study**

If abnormality of intestinal immune function is detected through this study, the information will be applied for better treatment of patients as much as possible.

**Privacy protection**

All serum samples will be labeled with serial number and serum collection date without patient’s name.

**Informed Consent**

The study purposes and procedures were provided in written form, and informed consent was obtained from all patients and normal subjects before performing any study procedures according to the Declaration of Helsinki

**Funding and Conflict of Interest**

Individual organizations will support this study independently

**Payment to patients participating in study**

No gratuity to patients and others

**Ethics Committee**

Independent Ethics Committee, Asahikawa Medical University

**Clinical Study Registration Number (UMIN)**

Applied to University Hospital Medical Information Network (UMIN) Center

**UNIM No. 000012200**

**Attribution of research and development results**

The study data obtained in this study shall be shared by all contributed in this study. Data will be presented at Japanese Society of Rheumatology and then submitted to an appropriate scientific journal.

**Study Period**

Two years after the approval by the Independent Ethic Committee, Asahikawa Medical University.
